# Advancing brain immunotherapy through functional nanomaterials

**DOI:** 10.1007/s13346-024-01778-5

**Published:** 2025-01-09

**Authors:** Bhanu Nirosha Yalamandala, Thi My Hue Huynh, Hui-Wen Lien, Wan-Chi Pan, Hoi Man Iao, Thrinayan Moorthy, Yun-Hsuan Chang, Shang-Hsiu Hu

**Affiliations:** Department of Biomedical Engineering and Environmental Sciences, National Tsing Hua University, 300044 Hsinchu, Taiwan

**Keywords:** Glioblastoma, Nanomaterials, Immunotherapy, Blood-brain barrier, Brain tumor

## Abstract

Glioblastoma (GBM), a highly aggressive brain tumor, poses significant treatment challenges due to its highly immunosuppressive microenvironment and the brain immune privilege. Immunotherapy activating the immune system and T lymphocyte infiltration holds great promise against GBM. However, the brain’s low immunogenicity and the difficulty of crossing the blood-brain barrier (BBB) hinder therapeutic efficacy. Recent advancements in immune-actuated particles for targeted drug delivery have shown the potential to overcome these obstacles. These particles interact with the BBB by rapidly and reversibly disrupting its structure, thereby significantly enhancing targeting and penetrating delivery. The BBB targeting also minimizes potential long-term damage. At GBM, the particles demonstrated effective chemotherapy, chemodynamic therapy, photothermal therapy (PTT), photodynamic therapy (PDT), radiotherapy, or magnetotherapy, facilitating tumor disruption and promoting antigen release. Additionally, components of the delivery system retained autologous tumor-associated antigens and presented them to dendritic cells (DCs), ensuring prolonged immune activation. This review explores the immunosuppressive mechanisms of GBM, existing therapeutic strategies, and the role of nanomaterials in enhancing immunotherapy. We also discuss innovative particle-based approaches designed to traverse the BBB by mimicking innate immune functions to improve treatment outcomes for brain tumors.

**Graphical Abstract:**

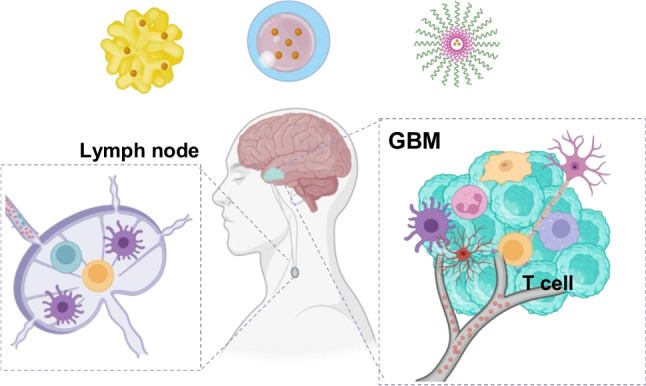

**Supplementary information:**

The online version contains supplementary material available at 10.1007/s13346-024-01778-5.

## Introduction

Glioblastomas (GBM) are the most common aggressive primary brain tumors that arise from glial cells and remain universally lethal. Despite its prevalence and aggressive nature, the effectiveness of traditional treatment options remains limited [1–3]. Over the past few decades, immunotherapy has become the attention of tumor treatment. It engages the body’s immune system to recognize and destroy cancer cells, which differs from traditional radiotherapy, chemotherapy, gene therapy, and surgery. The development of new drugs for GBM presents significant challenges, particularly due to the requirement for these therapies to cross the BBB. As such, the adjuvant of new delivery vehicles such as exosomes, that allow specific targeting of drugs at the tumor site may evolve to be a staple in GBM treatment. These nanocarriers can be engineered to enhance drug stability and bioavailability, ensuring a more effective delivery of therapeutic agents. Furthermore, their ability to incorporate both hydrophilic and hydrophobic drugs makes them versatile tools for addressing the complex nature of GBM [4–7]. Nevertheless, due to the highly immunosuppressive environment in GBM, it typically exhibits a weak immune response [8]. Moreover, the presence of the blood-brain barrier (BBB) also reduces the effectiveness of immunotherapy. Therefore, the effective immune response of GBM needs therapeutic agents not only to penetrate the BBB efficiently but also to relieve the potential immunosuppression of the GBM tumor microenvironment (TME) [9, 10].

The brain’s unique vascular system BBB as a major obstacle experiences endogenous immunosuppression and presents additional challenges to immunotherapeutic approaches. The immune system in the brain operates under unique principles through which access to the tumors is limited by BBB [11, 12]. Several promising types of immunotherapies are under investigation, including modulation of cytokines, dendritic cell (DC)-facilitated presentation of tumor-associated antigens, oncolytic viruses, adoptive transfer of modified immune cells, and checkpoint inhibition, including programmed cell death protein 1 (PD-1) and cytotoxic T lymphocytes (CTLs) [13, 14].

DC-based vaccines have indeed been a central aspect in the development of immunotherapies for brain tumors, especially in GBM [15, 16]. These vaccines aim to harness the potent antigen-presenting capabilities of DCs to stimulate a robust anti-tumor immune response. As specialized antigen-presenting cells (APCs), they have the distinct capacity to capture and process tumor antigens, which they present to T cells through major histocompatibility complex (MHC) molecules [17, 18]. This presentation is vital for initiating and influencing the adaptive immune response. When DCs present tumor antigens to T cells, particularly CD8^+^ cytotoxic T cells, they activate these T cells to identify and eliminate tumor cells expressing those specific antigens [19, 20].

In a therapeutic sense, DC vaccines are designed to stimulate tumor-specific CTLs that target and eliminate malignant cells [21, 22]. The key mechanism of action involves DCs processing and presenting tumor antigens to T cells, particularly CTLs, which then seek out and destroy tumor cells expressing those antigens. While progress in immunotherapy, the suboptimal impact of the influence of T-cell infiltration into the treatment of brain tumors persists as a significant barrier to effective immunotherapy. CTLs face significant challenges when attempting to infiltrate and eliminate tumor cells in brain tumors, largely due to the complex and hostile TME. Consequently, effective immunotherapy necessitates maintaining a careful balance between activating and sustaining enduring antitumor immunity [23, 24].

Many strategies have demonstrated great success in rodent models, but translating these findings into effective treatments for humans remains a significant challenge. For example, an effective strategy of immunotherapy involves more than just the cytotoxic capability of immune cells but they also are trafficking to the tumor site and surviving in the TME [25, 26]. Trafficking the migration of immune cells into the brain is indeed one of the significant challenges. Overview of the existing challenges in brain tumor immunotherapy and recent developments in basic tumor immunology aimed at overcoming resistance to current treatments [27].

Nanoparticles (NPs) play a transformative role in regulating the TME by addressing its inherent challenges and enhancing therapeutic outcomes. Modifying the surface with specific ligands, polymers, or biomolecules can significantly improve their properties and performance. Functionalization with antibodies, peptides, aptamers, or small molecules allows nanomaterials to target tumor cells, immune cells, or specific receptors in the tumor microenvironment, improving precision [28, 29]. They can reprogram immune cells, such as shifting tumor-associated macrophages (TAMs) from a pro-tumorigenic to an anti-tumorigenic state and modulate hypoxia by delivering oxygen or catalytic agents. NPs target tumor vasculature for improved drug delivery, remodel the extracellular matrix to enhance immune cell infiltration, and neutralize immunosuppressive factors like TGF-β. Additionally, they enable pH-sensitive and ROS-mediated therapies, promote antigen release for immune activation, and act as carriers for chemotherapeutics or cancer vaccines. By integrating these capabilities, NPs overcome TME barriers and boost anti-tumor immunity, offering a potent approach [30–32].

Immune-actuated nanoparticles offer significant potential across various therapeutic applications by leveraging their ability to interact with and modulate the immune system. They can enhance the permeability of the BBB, facilitating the delivery of therapeutic agents to the brain for treating conditions such as brain tumors and neurodegenerative diseases [33, 34]. In cancer immunotherapy, nanoparticles can serve as carriers for immune checkpoint inhibitors, such as PD-1/PD-L1 blockers, to boost anti-tumor immunity and address immune evasion in tumors [35, 36]. Functionalized nanoparticles with immune stimulators, like CpG or TLR agonists, can activate immune cells, including dendritic cells and macrophages, making them useful in both cancer immunotherapy and the treatment of infectious diseases [37, 38]. Additionally, nanoparticles can be engineered to modulate the tumor microenvironment, making it more conducive to immune cell attack, thus enhancing tumor immunotherapy and immunomodulation, particularly in immunosuppressive tumors [39, 40]. They also facilitate antigen release and promote immune cell activation, which is crucial for cancer vaccines, oncolytic therapies, and the activation of dendritic cells [41, 42]. The integration of nanoparticles with other therapeutic approaches, such as chemotherapy [43], PTT [44, 45], radiotherapy [46, 47], and magnetotherapy [48, 49], enhances the efficacy of these treatments through synergistic effects. Furthermore, nanoparticles can deliver chemotherapeutic agents in a controlled manner, improving targeting while minimizing systemic toxicity, which is especially beneficial for treating drug-resistant tumors. This multifaceted role underscores the transformative potential of immune-actuated nanoparticles in modern medicine. (Table 1).

**Table 1:** Summarizing the different potentials of immune-actuated nanoparticles and their applications in various therapeutic strategies focusing on their ability to enhance immune responses

Potential of Immune-Actuated Nanoparticles	Description	Therapeutic Applications
Disrupting the BBB’s structure	Nanoparticles can enhance BBB permeability, allowing for the delivery of therapeutic agents to the brain.	Treatment of brain tumors, neurodegenerative diseases [33, 34]
Immune Checkpoint Therapy	Nanoparticles can carry immune checkpoint inhibitors (e.g., PD-1/PD-L1 blockers) to enhance anti-tumor immunity.	Cancer immunotherapy, overcoming immune evasion in tumors [35, 36]
Activation of Immune Cells	Functionalization of nanoparticles with immune stimulators (e.g., CpG, TLR agonists) activates immune cells (e.g., dendritic cells, macrophages).	Cancer immunotherapy, infectious disease treatment [37, 38]
Modulating Cellular and Tumor Microenvironment	Nanoparticles can be designed to alter the tumor microenvironment, making it more favorable for immune attack.	Tumor immunotherapy, immunomodulation, treatment of immunosuppressive tumors [39, 40]
Promoting Antigen Release and Immune Cell Activation	Nanoparticles can facilitate the release of tumor antigens, promoting T-cell activation and immune response.	Cancer vaccines, oncolytic therapies, dendritic cell activation [41, 42]
Integrating with Therapeutic Approaches	Nanoparticles can be combined with various treatments, enhancing their efficacy (chemotherapy, PTT, radiotherapy, magnetotherapy).	Combination therapy, synergistic cancer treatments [19, 129]
Chemotherapy	Nanoparticles can deliver chemotherapeutic agents in a controlled manner, improving efficacy and reducing systemic toxicity.	Targeted cancer treatment, drug-resistant tumors [43]
PTT	Nanoparticles can absorb light (e.g., NIR) and convert it into heat, inducing localized tumor cell death.	Cancer treatment, localized tumor destruction [44, 45]
Radiotherapy	Nanoparticles can enhance the effect of radiation by acting as radiosensitizers, improving tumor cell killing.	Tumor treatment, enhancing the efficacy of radiotherapy [46, 47]
Magnetotherapy	Magnetic nanoparticles can be directed to tumor sites using an external magnetic field, allowing for targeted therapy.	Targeted drug delivery, tumor imaging, localized hyperthermia [48, 49]

It has indeed revolutionized by offering significant improvements in drug delivery and treatment efficacy. Summarizing nanomaterial-based treatments for GBM, which could include a range of nanomaterials like carbon nanotubes (CNTs), gold NPs (Au NPs), dendrimers, nanogels, liposomes, and polymers. Each of these nanomaterials has unique properties that make them suitable for GBM therapy, including their ability to cross the BBB, target tumor cells or the tumor microenvironment, and deliver therapeutic agents as shown in Table 2. They enabled more precise targeting and controlled delivery of therapeutic agents with featured enhanced accumulation, penetration, internalization, and controlled release [50]. Drug delivery systems (DDS) play a vital role in addressing these challenges and ensuring that each drug in a combination therapy reaches its target effectively. For example, DDS can be engineered with specific ligands that target receptors on the BBB. This functionalization improves the ability to cross the BBB and penetrate the targeted tissues. By conjugating ligands that selectively bind to receptors on the endothelial cells of the BBB (such as transferrin or glucose transporters), DDS can facilitate receptor-mediated endocytosis. This process permits the nanocarriers to be transported across the BBB more effectively than non-targeted systems. Targeting ligands can induce changes in the BBB’s permeability, allowing the DDS to transport not only the carrier but also the therapeutic agents it carries across the barrier [51].

**Table 2:** Nanomaterial-based approaches for glioblastoma GBM therapy

Materials	Physicochemical properties	Targeting ligands	Functions	Major findings in GBM	Outcome
Carbon nanotubes	With high surface area, strength, and conductivity, their functionalization enables diverse applications	Folate, RGD peptides, aptamers, transferrin, antibodies, PEG [60, 61]	-Chemoresitant GBM -Migration inhibition of GBM -Immune stimulation	-Retention within tumors which increases the efficacy of GBM treatment in mice -Decreased NF-κB activation in GBM cells, immunostimulatory property, and migration of GBM cells -TME based cancer therapy, immune stimulation	-Tumors manipulate immune cells. [73] -CpG-conjugated CNTs inhibit brain tumor cell migration. [76] -Enable targeted drug delivery and enhanced imaging for effective treatment and real-time tumor monitoring in cancer theranostics. [77]
Gold NPs	High surface area, optical properties, and biocompatibility	Folate, RGD peptides, HER2 antibodies, transferrin, peptides, PEG [81, 82]	-pH-responsive treatments activate immune responses in GBM -T-cell activation	-Disruption of cell-cell interactions and T-cell infiltration. -Powerful ability for antitumor immune activation.	-pH-responsive dendrimers deliver dual drugs, boosting accumulation and efficacy in GBM. [93] -T cells target PD-L1-suppressed glioma, boosting PTT in GBM. [94] Spiky AuNPs boost biomolecule loading and T-cell activation via multivalent ligand-receptor binding. [96]
Dendrimers	Highly branched macromolecules with a high surface area	Folate, RGD peptides, mannose, peptides, antibodies, chitosan [103, 104]	Targeting TAMs in an immunocompetent GBM model enhances tumor immunotherapy.	-Immune reprogramming and immunocompetent model of GBM -Dendrimers alter the interactions in the GBM treatment. - TNF-α, IFN-γ, and IL-6 cytokines secretion, and improved PD-L1 expression of tumor cells.	-Dendrimer-triptolide reduced tumor burden more effectively than free triptolide. [109] -Glycosylated PAMAM dendrimers improve targeting specificity in GBM. [106] -Partially zwitterionic dendrimer-entrapped AuNPs boost dendritic cell functionality. [110]
Nanogels	A 3D network with high water retention and tunable size	Folate, RGD peptides, antibodies, peptides, hyaluronic acid [111, 112]	Chemoimmunotherapy in GBM utilizes antigen reservoirs to enhance T cell activation.	-Enhanced tumor permeability and prolonged blood circulation -The catalytic nanoreservoir at lung metastasis -Enhance drug delivery to GBM cells	-Single-dose nanovaccine in hydrogel induces local immune responses. [122] -DOX-based mannose nanogels trigger anti-tumor immunity. [122] -Adhesive nanoreservoirs use catalytic therapy and antigen capture. [123]
Liposomes	Hydrophilic core and customizable properties for targeted drug	Folate, RGD peptides, antibodies, transferrin, PEG [122, 113]	Targeted siRNA delivery and radio-immunotherapy	-Enhance drug delivery to GBM cells -The suppression of MDK by Plofsomes. -Immunogenic cell death and anti-tumor immunity.	- NF-κB inhibitors enhance drug delivery to glioma cells. [130] -Polymer-locked liposomes target siRNA and CRISPR-Cas to GBM. [131] - Neutrophil-carried immunoregulatory liposomes. [132]
Polymers	Adjustable macromolecules with customizable solubility and strength	RGD peptides, folate, antibodies, peptides, dextran, mannan, sugar molecules [135, 136]	Targeted siRNA delivery and radio-immunotherapy	-Antigen reservoir that retains autologous tumor-associated antigens. -Effective GBM chemo-immunotherapy. -Boosts the antitumor immunological effect.	- Near-infrared II nanoflakes activate dendritic cells, enhancing brain tumor immunotherapy. [141] - Redox-responsive micelles co-encapsulate immune inhibitors and chemotherapeutics for synergistic GBM therapy. [142] - Target neutrophils for drug delivery, track with NIR-II, and use PTT with nitric oxide to promote tumor death and immune activation. [143]

DDS can be designed as antibody-drug conjugates that consist of an antibody linked to a drug. The antibody specifically targets antigens expressed on the cells of the BBB. This targeted method confirms that the drug is administered precisely where it is required, increasing its therapeutic effect while minimizing off-target toxicity [52]. Furthermore, Nanoparticles (NPs) can be coated with polymers that enhance their ability to interact with the BBB. For instance, polyethylene glycol (PEG) can be used to prolong circulation time and improve stability, while targeting ligands ensure efficient BBB penetration [53]. Functionalized DDS can be designed to co-deliver multiple agents (chemotherapeutics, immunomodulators) that work synergistically to enhance treatment efficacy while ensuring that each drug reaches its target site [54].

**Fig. 1:**
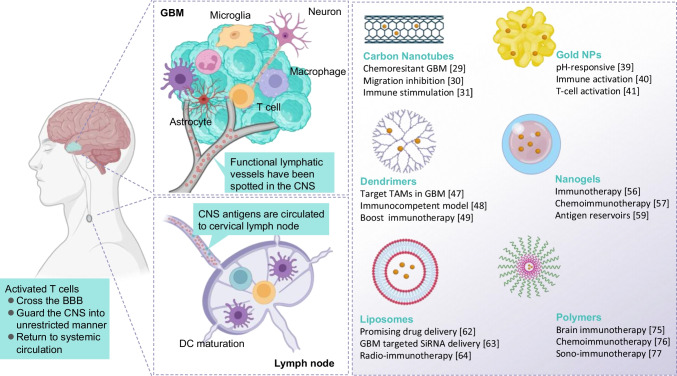
Schematic illustration of Functional nanomaterial-based immunotherapeutic approaches in GBM

The tight junctions in the BBB play a critical role in maintaining the selective permeability of the central nervous system (CNS) as shown in Fig. 1. These tight junctions are formed by complex protein interactions, including claudins, occludins, and junctional adhesion molecules, which are anchored to the cytoskeleton through adaptor proteins. This structural arrangement ensures a robust seal between endothelial cells lining the brain’s capillaries, effectively preventing the paracellular transport of solutes, pathogens, and immune cells from the bloodstream into the brain. The evolving understanding of the immune environment within GBM and insights into the presence and function of immune components. Various types of APCs contribute to the GBM tumor’s immune microenvironment including microglia, macrophages, astrocytes, and traditional APCs like DCs. Recent advances in various nanoscale drug delivery systems designed to enhance immunotherapy in GBM. NPs such as CTSs, AuNPs, dendrimers, nanogels, liposomes, and polymeric nanocarriers have extensively been used as drug delivery vehicles for site-specific treatments.

## Engineering carbon nanotubes (CNTs) for enhancing brain immunotherapy

CNTs have garnered significant interest as drug candidates in nanomedicine due to their excellent mechanical, and physicochemical properties. This novel nanomaterial has garnered substantial scientific interest over the past decade [55, 56]. CNTs offer several potential uses in cancer, including drug delivery, imaging, and combination therapy [57].

They can be utilized as a carrier for drug delivery systems, enabling targeted and controlled release. Owing to the exceptionally high surface area, they allow for the efficient loading of therapeutic agents. The robust structure of CNTs provides mechanical strength and stability, rendering them suitable for drug delivery in challenging environments. Furthermore, carbon nanotubes (CNTs) can be functionalized through several strategies to enhance their biomedical applications. Covalent functionalization with carboxyl or amine groups improves their solubility and biocompatibility, facilitating their integration into biological systems. Non-covalent functionalization using surfactants or hydrophilic polymers, such as polyethylene glycol (PEG), enhances their stability and prevents undesirable aggregation. Functionalized CNTs also exhibit enhanced dispersion in physiological environments, which is critical for reducing aggregation and improving bioavailability. These properties are essential for efficient therapeutic and diagnostic applications. Moreover, their unique ability to penetrate cellular membranes makes them highly effective as vehicles for the intracellular delivery of drugs, offering targeted treatment options while minimizing systemic side effects [58, 59]. Additionally, CNTs can be modified with ligands [60, 61]. such as RGD peptides, antibodies, peptides, or antibodies that identify and attach to receptors on GBM cells. This targeted strategy improves the accumulation of drugs at the tumor location. CNTs can be engineered to release their payload in a controlled manner, either through changes in environmental conditions (e.g., pH, temperature) or by using stimuli-responsive materials. This allows for sustained therapeutic effects and reduced systemic toxicity. Due to their nanoscale size, CNTs can penetrate biological barriers, including the BBB, and also co-deliver multiple therapeutic agents, which may work synergistically to enhance treatment efficacy against GBM [62].

Furthermore, CNT combines with other therapeutics including PTT and PDT to potentially employ to damage cancer cells. The growing number of published reports using these systems highlights their increasing appeal in the biomedical field. This family includes several key groups of carbon NPs, including carbon dots (CDs), fullerenes, CNTs, nanodiamonds (NDs), and graphene [ 63]. Among the several kinds of carbon nanomaterials, fullerenes, and CNTs have attracted the most attention. The unique properties of CNTs, including their elevated aspect ratio, versatile surface chemistry, and tunable morphology and structure, are highly beneficial for targeting and transporting antigens through cell membranes. These properties contribute to enhanced target specificity and efficiency [64, 65].

CNTs can potentially overcome major challenges in the vaccine development and immunotherapy applications in GBM therapeutics. These applications develop a consideration of brain tumor interactions with the immune system. Although previous studies have revealed the potential to enhance the immune response as adjuvants, we examine the recent developments in CNT-based immunological research, emphasizing current insights into the therapeutic effectiveness and mechanisms of brain tumor immunotherapy [66]. Engineering CNTs for immunotherapeutic applications involves modifying their structure, surface chemistry, and functionalization to optimize their communication with the immune system, enhance therapeutic delivery, and improve biocompatibility [67].

Recent research has demonstrated that GBM cells are mechanosensitive, meaning they respond to the mechanical properties of their environment [68]. This finding has significant implications for understanding tumor progression and developing new therapeutic strategies. Developing a nanoscale mechanical approach to treat GBM using nanomaterials is an exciting and emerging strategy that leverages the mechanosensitivity of GBM cells. It can influence various behaviors, including proliferation, migration, and invasion. Understanding these responses can inform the design of nanomaterials that exploit these characteristics for targeted drug delivery. Mechanical perturbation has indeed been explored as a method to target cancer cells, including GBM. This strategy leverages the sensitivity of GBM cells to mechanical forces. By applying external or internal mechanical stresses, it is possible to disrupt cancer cell integrity or trigger mechanotransduction pathways that can lead to cell death or increased susceptibility to treatment [69].

**Fig. 2:**
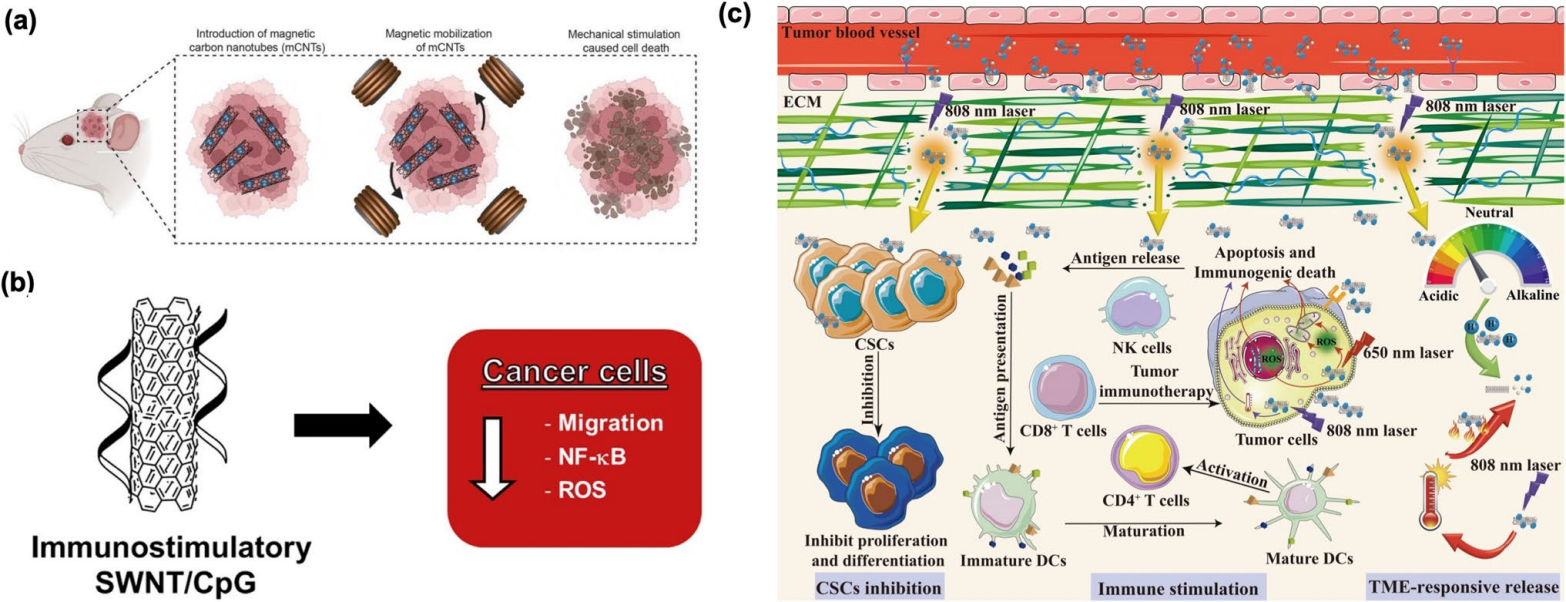
**(a)** mCNTs can induce cell death when subjected to a rotating magnetic field. The magnetic properties of mCNTs allow them to be directed and activated in the tumor environment. When exposed to the rotating magnetic field, the mCNTs exert torque on the cancer cells, mechanically stressing them and causing damage [73]. **(b)** The immunostimulatory CpG molecules attached to carbon nanotubes (CNTs) have been shown to specifically inhibit the migration of brain tumor cells, particularly glioblastoma (GBM), without affecting the movement of macrophages. These CpG-functionalized CNTs retain their ability to stimulate immune responses while specifically targeting GBM cells [76]. **(c)** A carbon nanotube-based drug delivery system (DDS) targeting the tumor microenvironment (TME). The design illustrates key interactions and functions of CNTs within the TME for enhanced cancer therapy [77]

For example, magnetic field-actuated nanomaterials represent a promising strategy for mechanically targeting cancer cells, including GBM. This approach works by applying an external magnetic field to manipulate magnetically responsive nanomaterials that have been delivered to the tumor site [70, 71]. The key mechanism involves inducing mechanical stress that disrupts the integrity of the cancer cell membrane, prominent to cell death in GBM cells, which had been preincubated with magnetic NPs. The use of a magnetic field to manipulate magnetic NPs (MNPs) within tumors has shown promise in suppressing GBM growth. Likewise, the mobilization of mitochondria-targeting MNP chains using a magnetic field has shown considerable effectiveness in inhibiting GBM growth in preclinical models, particularly in mice [72]. This approach combines targeted therapy with mechanical perturbation, focusing specifically on disrupting mitochondrial function in GBM cells. Indeed, while magnetic field–controlled nanomaterials have shown promise in mechanosensitive tumors like GBM, their potential in overcoming chemoresistant tumors which are a major cause of relapse and patient mortality remains largely unexplored. Wang et al. [73] developed a spatiotemporally controlled rotating magnetic field that can be used to activate magnetic carbon nanotubes (mCNTs) to generate mechanical forces, which, in turn, induce cell death in GBM cells (Fig. 2a). They demonstrated surface functionalization of mCNTs with CD44 antibodies significantly enhances their enrichment and retention within tumor tissues, which increases the efficacy of GBM treatment in mice.

The migratory phenotype of GBM cells has been linked to treatment resistance and decreased apoptosis, which poses significant challenges in effectively managing this aggressive type of brain tumor. The idea that migration inhibitors may interfere with pro-tumor pathways in GBM is promising. Research integrating cancer migration inhibition with immunotherapy is still in its early stages, and several gaps need to be addressed [74]. Since the majority of migration inhibitors have primarily been evaluated using cancer cells, their effects on immune cells remain largely unexplored [75].

Alizadeh et al. [76] demonstrated the single-walled carbon nanotubes (SWNTs) non-covalently modified with CpG (SWNT/CpG) preserve the immunostimulatory properties of CpG and hinder the migration of GBM cells while leaving macrophages unaffected (Fig. 2b). Importantly, this inhibition doesn’t impact cell viability or proliferation. The research showed that SWNT/CpG also specifically diminishes NF-κB activation in GBM cells, while concurrently stimulating macrophages via the TLR9/NF-κB pathway. The reduced migration of GBM cells was linked to a selective decrease in intracellular reactive oxygen species (ROS) levels, suggesting the antioxidant-related mechanism is likely responsible for the observed effects.

Tang et al. [77] highlight recent uses of CNTs in TME-based cancer therapy have harnessed their unique properties to improve treatment outcomes (Fig. 2c). Various strategies have emerged, including the use of CNTs for remodeling the extracellular matrix (ECM) to facilitate better drug penetration, targeting tumor vasculature to disrupt blood supply, and stimulating the immune system to boost anti-tumor responses. Additionally, CNTs have been employed to inhibit cancer stem cells (CSCs), significantly to tumor growth and recurrence. Another notable application is TME-responsive drug release, where CNTs are intended to release therapeutic agents in response to specific stimuli in the TME, ensuring precise and controlled treatment.

This work opens several exciting avenues of magnetic field parameters that could refine the mechanical force application ensuring maximum therapeutic efficacy. Moreover, combining this approach with immunotherapy could synergize the effects and offer a comprehensive treatment strategy. Overall, the platform holds significant promise as a dual-action therapeutic strategy by selectively inhibiting GBM cell migration while promoting macrophage activation. Furthermore, the specific role of ROS modulation in GBM cell migration warrants deeper investigation, as it may open up new avenues for targeting similar oxidative stress profiles. By modulating both cancer cell behavior and immune activity, we can develop more sophisticated, targeted approaches to treating highly aggressive cancers like GBM.

## Enhancing immunotherapy efficacy in GBM with Gold NPs (AuNPs)

Infiltration of T cells into the brain remains a key challenge in GBM immunotherapy [78]. The targeting of zwitterionic carriers using TME-sensitive charge conversion has been displayed in enhancing the accumulation of drugs in GBM. Responsive nanomedicine has emerged as a powerful tool for delivering chemotherapy drugs and also for addressing the complexities of improved penetration of these delivery systems, modification of tumor immunogenicity, and T cell activation.

AuNPs have developed as a powerful asset in immunotherapy owing to their stability, biocompatibility, and ability to be functionalized for targeted delivery. When used for antigen delivery, AuNPs can specifically target APCs, including DCs, enhancing the immune response and overcoming several challenges associated with traditional vaccine delivery systems [79, 80]. Additionally, AuNPs can be modified with polymer coatings or thiol groups to enhance their stability, while targeting ligands [81, 82] like antibodies, RGD peptides, folate, aptamers, or transferrin can be conjugated to direct them toward specific cells. Therapeutic agents, including chemotherapeutic drugs, can also be attached for controlled release. Surface modification of AuNPs further improves cellular uptake, extends circulation time, and reduces off-target effects, making them ideal for precise drug delivery. Moreover, when incorporated into combination therapies with immunotherapies and radiotherapies, AuNPs have shown potential for synergistically improving anti-tumor efficacy. By optimizing their surface properties, AuNPs can overcome biological barriers, such as blood vessels and the extracellular matrix, facilitating the targeted delivery of immunomodulators or antigens to DCs and T cells [83, 84].

In general, the physicochemical characteristics of AuNPs can significantly influence their biocompatibility, biodistribution, and functionality. Smaller AuNPs typically exhibit improved ability to traverse the BBB by passing *via* the gaps between astrocyte end-feet and capillary endothelium, allowing them to extravasate into brain tumor tissue [85]. As brain tumors progress, the BBB undergoes structural and functional impairment, leading to a “leaky” BBB in and around the tumor [86]. AuNPs in the 100 nm range can take advantage of these leaky regions to penetrate the tumor through a strategy called the enhanced permeability and retention (EPR) effect [87]. Additionally, AuNPs smaller than the safe renal clearance threshold (approximately 6 nm) not only exhibit enhanced targeting ability of GBM but also be rapidly excreted via the renal route. In contrast, larger AuNPs have shown persistence in the liver and spleen of mice for up to six months [88].

Despite recent advantages, the challenge of penetrating the tumor-associated BBB remains significant, with its tightly packed endothelial cells, tight junctions, and active efflux transporters, especially in cases like GBM [89]. BBB is highly selective, and one of its most significant limitations is its restriction on the passage of molecules larger than 500 Da. This means that 98% of small-molecule drugs and almost all large biomolecules are blocked from entering the brain [90]. Designing NPs with BBB-bypassing capabilities presents a promising strategy to address the challenges presented by BBB, particularly for delivering therapeutic agents to the brain. The use of viral mimicry, including incorporating rabies virus glycoprotein (RVG), has emerged recently as an innovative strategy to circumvent the BBB and deliver therapeutic agents to the central nervous system (CNS) [91]. RVG specifically interacts with nicotinic acetylcholine receptors found on neuronal cells, aiding in the transport of therapeutic molecules into the brain. This method has attracted considerable attention as a targeted strategy for treating brain disorders by improving drug delivery to the CNS [92].

**Fig. 3:**
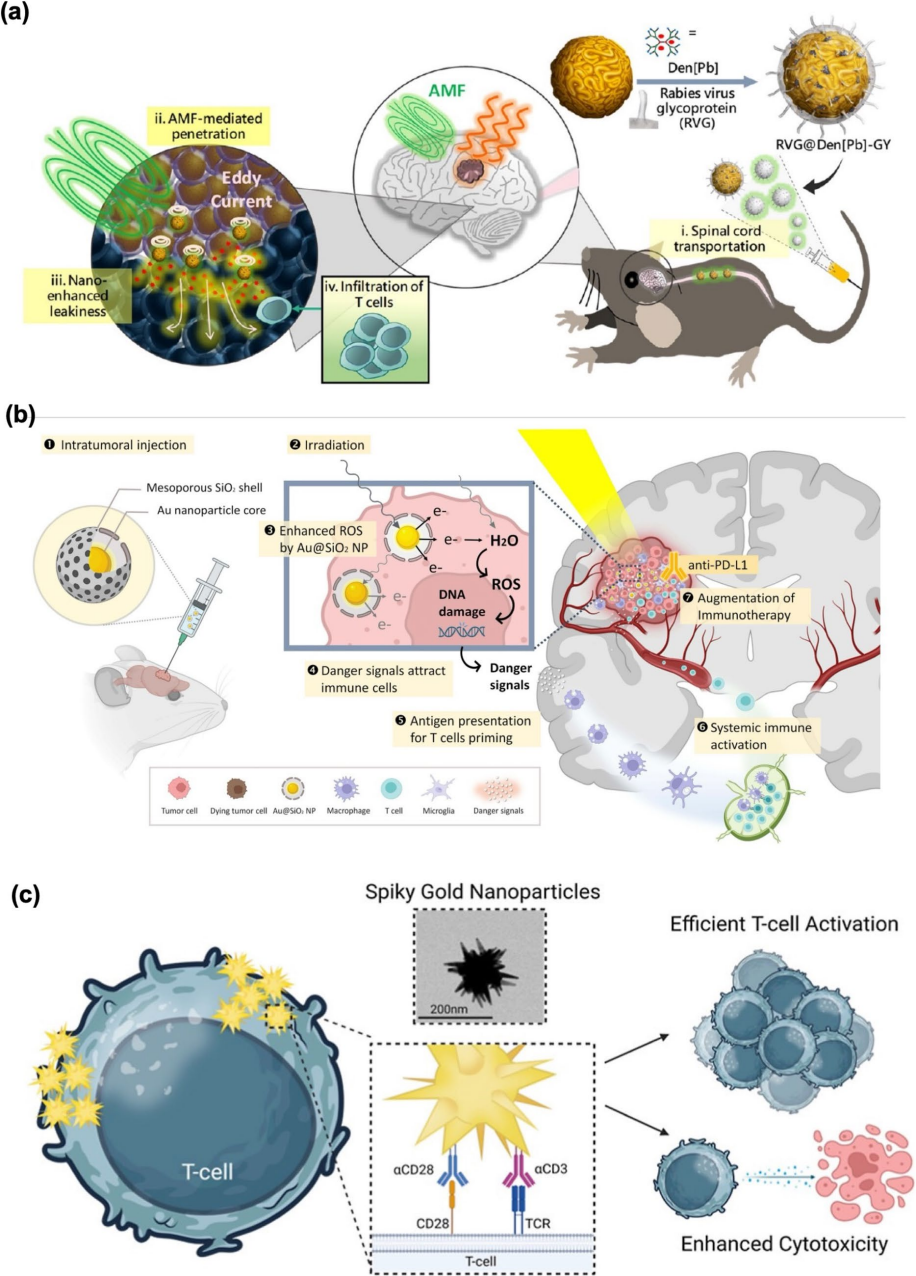
** (a)** The schematic depicts the hierarchical targeting of RVG-hybrids, which undergo an aggregation transition in slightly acidic conditions. This magnetoelectric disassembly of RVG-hybrids enhances the effective delivery of dendrimers and graphene quantum dots (GQDs) into the target area [93]. **(b)** Schematic illustration of a combined approach for treating GBM. It highlights the various components and techniques to enhance the effectiveness of the treatment strategy [96]. **(c)** Spiky AuNPs represent a nanoscale method designed to improve the activation of T-cells outside the body (ex vivo). This innovative approach enhances the immune response by facilitating better interactions between the NPs and T-cells [98]

For example, in our previous work, Cheng et al. [93] developed RVG-decorated hybrids (RVG-hybrids) that integrate pH-responsive dendrimers (pH-Den) with boron-doped graphene quantum dots (B-GQD) (Fig. 3a). These hybrids are designed to co-deliver two drugs: palbociclib (Pb), which is encapsulated within the dendrimers, and doxorubicin (Dox), which is linked to the B-GQDs, specifically targeting orthotopic GBM. The hybrids naturally accumulate in the acidic tumor microenvironment and possess the capability to traverse the BBB via partial spinal cord transport. In vivo studies demonstrated that the hybrids effectively target brain tumors, and their HFMF-triggered penetration improves drug distribution for synergistic therapeutic effects.

Furthermore, radiation therapy is a cornerstone in the treatment of GBM. It works by inducing intracellular damage primarily through the generation of ROS. The integration of AuNPs into radiation therapy is an innovative approach and is enhanced by acting as radiation sensitizers. When AuNPs are localized within or near tumor cells, they can absorb and scatter radiation more effectively than surrounding tissues. This leads to an increase in localized radiation dose at the tumor site, resulting in enhanced ROS generation and, consequently, increased cell damage. Here’s how Au NPs enhance radiation therapy through the generation of reactive oxygen species (ROS) [94]. The high atomic number and electron density lead to the generation of additional secondary electrons, which contribute to enhanced radiation effects of AuNPs. The emission of secondary electrons, particularly Auger electrons, and photons resulting from the photoelectric effect of AuNPs significantly contributes to enhanced ROS generation and sensitization effects during radiation therapy [95].

To further amplify the immunogenic effects, Chen et al. coated AuNPs with a mesoporous silica shell (SiO_2_), combined with X-ray irradiation (XR), to improve the effectiveness of the anti-PD-L1 antibody (α-PD-L1) in treating brain tumors (Fig. 3b) [96]. In this study, the SiO_2_ shell plays a dual role, enhancing immune stimulation and providing sustained radiosensitization. They studied the effects of Au@SiO_2_ NPs under radiation, observing increased oxidative stress, recruitment of immune cells, and induction of immunological cell death. This system demonstrated a strong capacity to activate antitumor immune responses, offering a promising new approach for treating GBM. Additionally, the combination of AuNPs with irradiation resulted in the modulation of the tumor microenvironment (TME), further promoting immune cell infiltration and enhancing the therapeutic effects. These findings suggest that AuNP-based strategies could provide a potent and versatile platform for improving the treatment outcomes of GBM.

For instance, investigations into the impact of AuNPs in various shapes, including nanospheres, nanocubes, and nanorods, on DCs have shown that nanoparticle shape significantly influences immune signaling pathways. This relevance of particle shape also applies to aAPCs, where it has a substantial impact on T-cell activation. Anisotropic aAPCs, such as hexapod, ellipsoidal, and tubular-shaped particles with high aspect ratios, have been demonstrated to be more effective than their isotropic, spherical T cell activation. This enhanced activation is largely attributed to their extended surface area. Anisotropic AuNPs, including gold nanostars and spiky AuNPs, have revealed superior benefits from therapeutic deliveries to PTT and Raman spectroscopy [97]. These anisotropic NPs demonstrate better targeting efficiency due to their tunable ligand density and shape-specific advantages.

While anisotropic AuNPs have been shown to modulate immune responses effectively in macrophages and DCs, there is a notable gap in understanding how these NPs interact with other immune cells, particularly T cell lymphocytes. Motivated by these findings, Esmaeili et al. [98] explore the use of spiky AuNPs as artificial APCs for ex vivo T-cell activation by presenting costimulatory ligands on their surface (Fig. 3c). The spiky design provides a larger surface area and positive curvature on the branches, enabling a higher ligand density than spherical NPs. This structure promotes multivalent binding of ligands to T-cell receptors, enhancing T-cell activation. Results showed that spiky AuNPs outperformed conventional systems by achieving greater T-cell activation, larger expansion, less overstimulation and exhaustion, and increased release of inflammatory cytokines in both primary T-cells and CAR-T cells. These findings suggest that spiky AuNPs hold great potential for advancing next-generation T-cell-based immunotherapies.

The dual drug delivery, enhanced targeting, and BBB-crossing ability offer a powerful combination to combat the inherent resistance and invasiveness of GBM. Moving forward combining this approach with immunotherapy could offer new avenues for tackling GBM’s heterogeneity. Refining the size, shape, and surface modifications of AuNPs could enhance their biodistribution and targeting effectiveness. Combining radio-sensitization with immune activation to overcome the challenges of tumor resistance and immunosuppression. Highlight the potential of spiky AuNPs to revolutionize T-cell-based immunotherapies. This platform could be further optimized by exploring other surface modifications, including the incorporation of additional stimulatory or inhibitory signals to precisely regulate T-cell activity.

## Dendrimer delivery improves antitumor immune signaling

Dendrimer-facilitated nanomedicines have demonstrated considerable promise in addressing brain tumors, owing to their highly branched, tree-like architectures that facilitate the accurate delivery of therapeutic agents. Their distinctive characteristics render them excellent candidates for tackling challenges like traversing the BBB and specifically targeting cells within the brain. As multivalent, monodisperse, and precisely engineered macromolecules, dendrimers serve as optimal nanoplatforms for the advancement of targeted drug delivery systems [99, 100]. Dendrimers can be functionalized with various chemical groups to improve their targeting, stability, and drug delivery capabilities. Surface modifications with ligands like folate, RGD peptides, or antibodies allow dendrimers to specifically target tumor cells. Additionally, they can be loaded with therapeutic agents such as drugs, siRNA, or imaging agents for controlled release. The dendrimer structure enables efficient encapsulation of both hydrophobic and hydrophilic compounds, while surface functionalization can enhance biocompatibility and reduce toxicity. This versatile design allows dendrimers to serve as effective carriers for combination therapies, enhancing the treatment efficacy by delivering multiple agents simultaneously. Furthermore, dendrimers can be engineered to respond to external stimuli, such as pH or temperature changes, to release their payload in a controlled manner, providing additional therapeutic benefits [101–104]. The small sizes and flexible surface modifications of dendrimers make them particularly suitable for accumulating the BBB and targeting GBM. Tumor-associated macrophages (TAMs) are crucial contributors to the advancement and severity of GBM. Research has shown that both infiltrating and resident macrophages/microglia within GBM can be altered by GBM cells, and their presence is positively associated with tumor grades. This interaction has significant implications for tumor progression and is closely linked to the severity of the disease [105].

Earlier research has shown that polyamidoamine (PAMAM) generation 4 hydroxyl-terminated dendrimers are capable of traversing a compromised BBB following systemic administration, allowing them to specifically target activated microglia and macrophages [107]. This feature makes them highly promising for treating various neurodegenerative diseases, where neuroinflammation plays a key role. These dendrimers are especially effective for the targeted delivery of therapeutic agents, providing accurate treatment. They also show promise in enhancing therapies for disorders like Alzheimer’s, Parkinson’s, and multiple sclerosis [108].

**Fig. 4:**
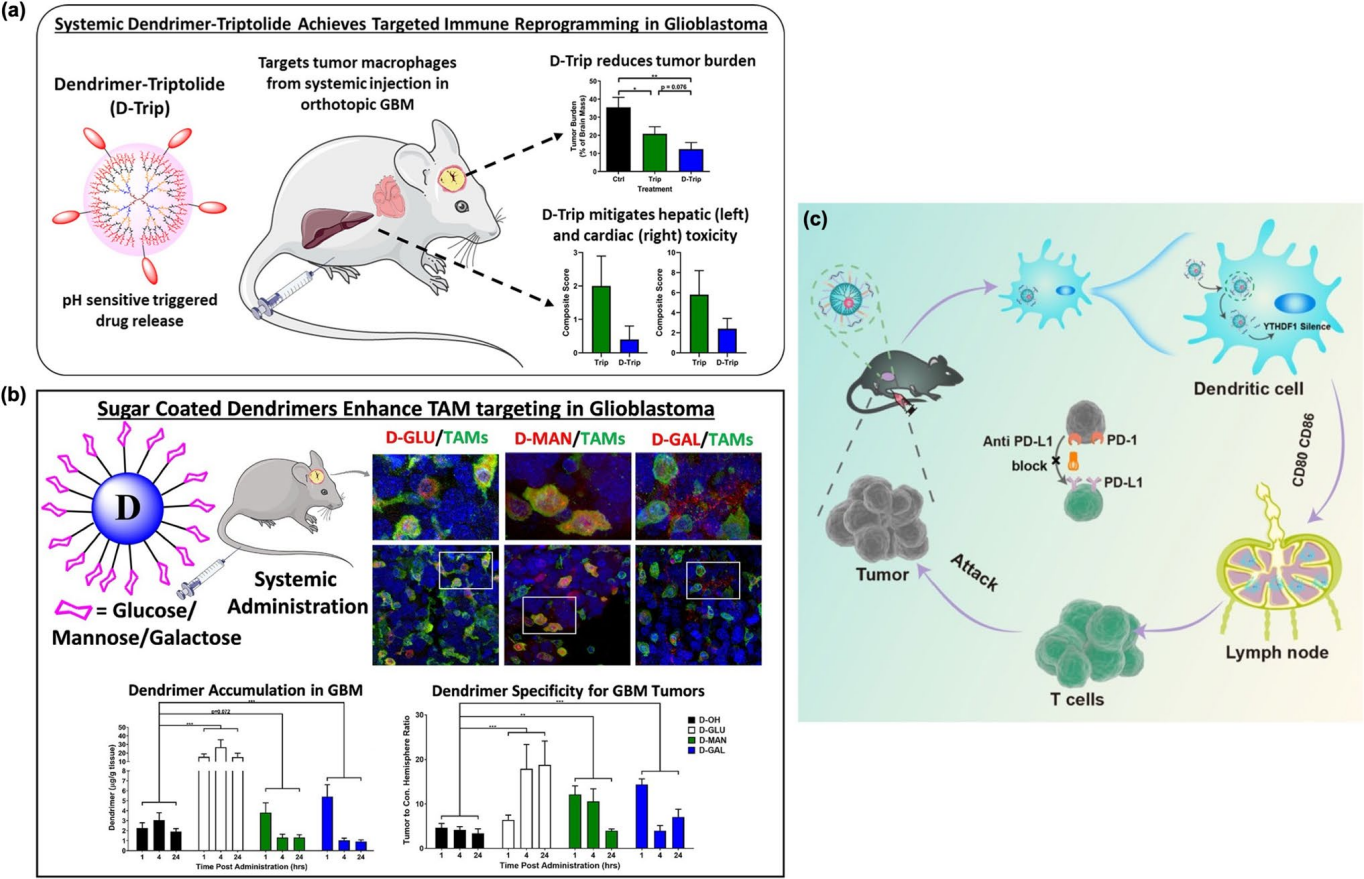
**(a)** The systemic delivery of triptolide using dendrimers specifically targets tumor-associated macrophages, enhancing the anti-tumor effectiveness while minimizing systemic toxicity in GBM treatment [106]. **(b)** The glycosylation of PAMAM dendrimers greatly enhances their ability to specifically target TAMs in GBM [109]. **(c)** Dendrimer-entrapped AuNPs enhance the effectiveness of tumor immunotherapy [110]

In the context of GBM, these dendrimers have been shown to successfully infiltrate solid brain tumors and specifically target TAMs in an orthotopic gliosarcoma model after systemic administration. Liaw et al. [109] developed a dendrimer-triptolide conjugate aimed at targeted systemic delivery to solid brain tumors, particularly focusing on TAMs (Fig. 4a). They employed a highly efficient click chemistry approach for synthesizing these conjugates. The study included in vitro investigations to assess immune reprogramming, along with in vivo evaluations of their effects in an orthotopic, immunocompetent GBM model.

In the context of brain tumors, Sharma et al. [106] proposed PAMAM dendrimers with sugar molecules to improve targeting specificity in GBMs, enabling them to broaden their specificity from TAMs to directly target cancer cells as well (Fig. 4b). They investigated how surface modifications with sugars such as glucose, mannose, or galactose influence targeting GBM. Their results demonstrated that these glycosylated PAMAM dendrimers improve targeting specificity in GBMs and significantly modifies the dendrimers’ interactions, thereby improving their efficacy in GBM treatment.

Ouyang et al. [110] developed genetically engineered DCs by silencing the expression of YTHDF1, a critical protein involved in RNA m6A methylation, utilizing a partially zwitterionic dendrimer entrapped AuNPs to enhance immunotherapy (Fig. 4c). The resulting nanoplatforms exhibited good stability and biocompatibility, along with high efficiency in delivering YTHDF1 siRNA at an optimized N/P ratio of 8:1. In vitro studies revealed that DCs transfected with the MDNP/siYTH polyplexes effectively downregulated YTHDF1 at both gene and protein levels while significantly upregulating maturation markers CD80 and CD86. When combined with the immune checkpoint blocker aPDL1, this strategy further enhanced T cell-based antitumor immunotherapy, as demonstrated by increased antitumor immune responses characterized by a marked presence of cytotoxic and effector T cells in the spleen and tumor. Additionally, there was elevated secretion of cytokines such as TNF-α, IFN-γ, and IL-6 in serum, alongside increased PD-L1 expression.

This work opens up new possibilities for combination therapies, where dendrimer-triptolide conjugates could be used alongside other immune-modulating agents, such as checkpoint inhibitors or cancer vaccines, to further boost anti-tumor immunity. By specifically delivering triptolide to TAMs and reprogramming the tumor microenvironment. Further, combining different sugar modifications to create multifunctional dendrimers capable of targeting multiple cell types within the tumor microenvironment. This could be particularly effective in combination therapies, where the dendrimer could simultaneously deliver chemotherapeutics to cancer cells and immunomodulatory agents to TAMs, amplifying the therapeutic response. By targeting RNA modifications, specifically m6A methylation through YTHDF1 silencing, we can significantly enhance the antigen presentation and maturation capabilities of DCs, rendering them more effective in priming T cells for a robust immune response. Combining checkpoint inhibitors strengthens these effects, resulting in a more comprehensive and lasting antitumor immunity. The implications of this study extend beyond the direct application to DCs and tumor immunotherapy. A deeper understanding of how RNA modifications regulate immune responses unveils new opportunities for therapeutic interventions in various immune cell types and disease contexts. Furthermore, utilizing a dendrimer non-viral vector for siRNA delivery is particularly promising, as it provides a highly efficient and non-toxic approach to gene editing, which could be adapted for targeting other pathways in cancer and immune modulation.

## Mechanism of bioengineered nanogels for immunomodulatory therapeutics

Nanogels designed for passive targeting can enhance the ability to penetrate the BBB, thereby facilitating increased drug accumulation within the tumo. Nanogel-based immunotherapy has garnered considerable interest owing to its improvement in the delivery and efficacy of immunotherapeutic agents. They can be functionalized with various ligands, polymers, or targeting moieties to enhance their drug delivery and therapeutic efficacy [111, 112]. Functionalization with molecules allows nanogels to target cancer cells. Additionally, surface modifications can improve their stability, control drug release in response to environmental stimuli (e.g., pH or temperature), and increase their biocompatibility. They are highly versatile, crosslinked polymeric particles of drugs, antigens, or biomolecules. Their unique properties make them especially suitable for cancer immunotherapy. Multifunctional nanogels, a significant advancement in nanomedicine, offer a powerful approach to cancer immunotherapy by re-educating and activating the immune system. These nanogels can deliver multiple therapeutic agents, such as antigens, immune adjuvants, or immunomodulators, in a controlled manner, enhancing their effectiveness in targeting tumors. Moreover, their ability to encapsulate both hydrophobic and hydrophilic agents expands their utility in various therapeutic contexts. Additionally, the use of nanogels in combination with immune checkpoint inhibitors and other therapeutic modalities could further boost their antitumor efficacy [113–118].

Owing to their highly cross-linked and hydrated polymer structure, nanogels can endure shear forces and the presence of serum proteins in the bloodstream, making them well-suited for systemic delivery in cancer immunotherapy. Their prolonged circulation time, enhanced stability, and resistance to degradation in the bloodstream increase their likelihood of accumulating in tumors, often facilitated by the EPR effect. The substantial specific surface area of nanogels provides a flexible platform for incorporating stimuli-responsive functional groups, allowing for various physical and chemical modifications that significantly enhance their therapeutic potential in immunotherapy. Such modifications can improve the targeting of specific immune cell subpopulations, boost drug bioavailability, and reduce adverse events [119].

In contrast to free antigens, nanogels improve the antigen cross-presentation to CD8^+^ T cells and minimize the effects of adjuvants, thereby eliciting a robust anti-tumor immune response. This improvement is attributed to their optimized nanosize and the capability to modify surface ligands and receptors, allowing for both passive and active targeting of APCs [120, 121].

**Fig. 5:**
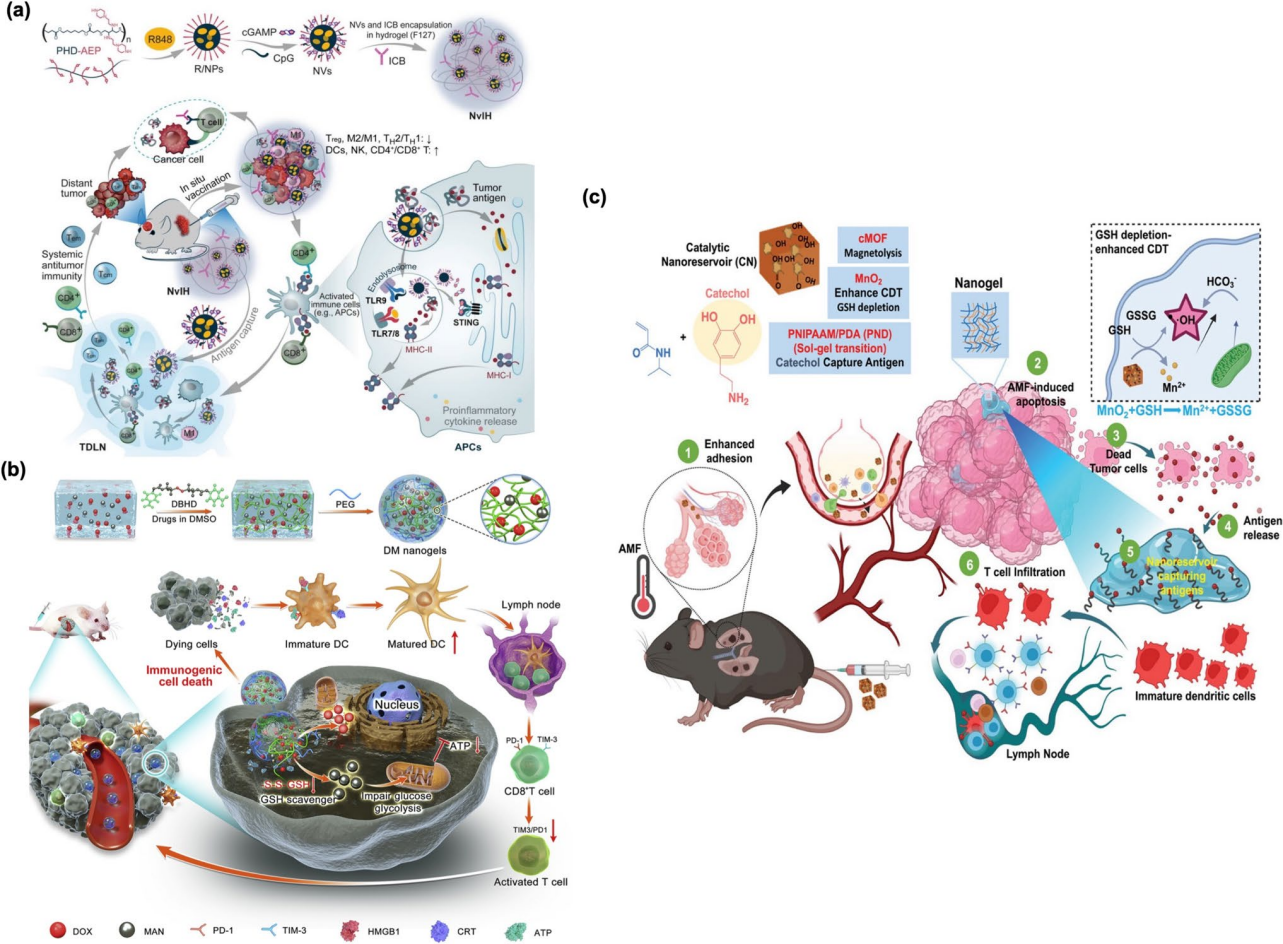
** (a)** The in situ vaccination using a single-dose NvIH effectively decreased immunosuppression within the TME and improved the overall antitumor immune response. This method resulted in an abscopal effect, where the treatment not only affected the targeted tumor but also had a beneficial impact on distant tumor sites [113]. **(b)** The schematic illustrates a bioresponsive mannose nanobackpack designed for cancer immunotherapy, which enhances the induction of immunogenic cell death (ICD) using DOX. The synthesis process of these drug-loaded nanogels, along with a simplified mechanism of chemo-immunotherapy aims to inhibit tumor growth by enhancing the induction of immunogenic cell death [122]. **(c)** The schematic depicts an injectable adhesive catalytic nanoreservoir (CN) engineered to function as an antigen reservoir for enhancing immunotherapy. The in situ gel formation aids in retaining the released antigens, thus supporting sustained immune stimulation and inhibiting tumor metastasis at the tumor site [123]

Cheng et al. [122] introduced a single-dose injectable hydrogel termed (nano vaccines + ICBs)-in-hydrogel (NvIH), specifically engineered for robust immunotherapy targeting large tumors and incorporating an abscopal effect (Fig. 5a). This thermo-responsive hydrogel co-encapsulates immune checkpoint blockade (ICB) antibodies alongside innovative polymeric NPs that carry three immunostimulatory agonists aimed at Toll-like receptors 7, 8, and 9 (TLR7/8/9) as well as the stimulator of interferon genes (STING). The NvIH design signifies a major advancement by addressing several critical challenges commonly encountered in immunotherapy.

Ma et al. [113] developed single-dose nanovaccine, DOX-loaded mannose nanogels (DM NGs) to explore the mechanisms underlying chemo-immunotherapy induce local immune responses (Fig. 5b). The DM NGs demonstrated notable micellar stability, selective drug release, and extended survival times, which were attributed to improved tumor permeability and prolonged circulation time in the bloodstream. Their findings indicated that DOX delivered via DM NGs could trigger a robust antitumor immune response by facilitating immunogenic cell death (ICD). Furthermore, the mannose released from the DM NGs exhibited significant synergistic effects against breast cancer.

Recently, in our prior research, Nirosha et al. [123] developed an adhesive catalytic nanoreservoir (CN) composed of manganese dioxide (MnO₂) and catechol-functionalized magnetic metal-organic frameworks (MOFs) for the capture of antigens (Fig. 5c). Upon intravenous injection, the CN accumulates at the tumor site via targeted margination, forming a gel in situ that effectively captures antigens. At the tumor location, the CN releases Mn²⁺ ions, which initiate redox reactions by depleting glutathione (GSH) and enhancing Fenton-like activity for chemodynamic therapy (CDT). In conjunction with hyperthermia, CDT facilitates the release of tumor-associated antigens, including neoantigens and damage-associated molecular patterns. The catechol-functionalized gels subsequently act as antigen reservoirs, delivering these autologous tumor-associated antigens to DCs to boost immune stimulation. This in situ-forming catalytic nanoreservoir effectively inhibited lung metastasis and improved survival rates through magnetothermal-induced antigen delivery.

By integrating nanovaccines with ICB within a hydrogel, we can achieve strong, localized immune activation. The demonstrated ability of NvIH to trigger both local and distant antitumor effects lays a solid foundation for future clinical applications, particularly for challenging tumors like GBM. Furthermore, the synergistic interaction between chemotherapy and immunotherapy presents a dual advantage by addressing the tumor directly while simultaneously stimulating the immune system. The development of DOX-based mannose nanogels marks a significant improvement in chemo-immunotherapy. By leveraging the properties of nanoscale platforms, we can enhance drug delivery, provoke robust immune responses, and target the metabolic vulnerabilities of cancer cells. The application of the catalytic nanoreservoir at sites of lung metastasis showcases its potential in treating metastatic cancer. The magnetothermal capabilities of the CN not only facilitate the retention of antigens but also boost the overall effectiveness of the treatment by effectively inhibiting tumor growth and improving survival rates. This dual-targeted strategy, which focuses on tumors while also stimulating the immune system, presents a promising avenue for the development of next-generation cancer therapies.

## Liposomes on GBM treatments

Lipid-based NPs represent a promising strategy for treating GBM. These NPs can be customized to deliver therapeutic agents directly to tumor location, improving efficacy [124, 125]. Liposomes, a type of lipid-based nanovesicle, have become a key nanocarrier in immunotherapy because they can encapsulate cancer drugs, such as chemotherapeutics, immunomodulators, and antigens. By improving drug stability, bioavailability, and targeted delivery, liposomes significantly boost the effectiveness of cancer immunotherapies [126]. Functionalization with ligands like folic acid, transferrin, or RGD peptides allows liposomes to target specific receptors on tumor cells, improve cellular uptake, and reduce off-target effects. Surface modifications can also improve their stability, control drug release, and provide better therapeutic outcomes. Additionally, liposomes can be engineered to respond to specific stimuli within the tumor microenvironment, such as pH or enzymatic activity, to release their cargo in a controlled manner. This allows for a more localized and sustained therapeutic effect, minimizing systemic toxicity [127, 128].

Among the most common and effective immune agents in cancer immunotherapy are polypeptides, nucleic acids, and antibodies, each playing a unique role in modulating immune responses. These agents serve as highly promising carriers in cancer immunotherapy because of their ability to effectively deliver immune modulators and influence both humoral and cellular immune responses. These are closed bilayer structures formed from phospholipids, featuring a hydrophilic phosphate polar head and a hydrophobic lipid tail. This amphiphilic characteristic allows liposomes to encapsulate a wide variety of drugs, irrespective of their physicochemical properties. The primary advantages of liposomes in drug delivery are their biocompatibility and ability to encapsulate diverse types of molecules. Recently, a significant number of researchers have investigated and published findings on liposome-based nanoformulations for GBM. These studies underscore the potential of liposomes to enhance drug delivery to the brain, paving the way for improved therapeutic outcomes [129].

**Fig. 6:**
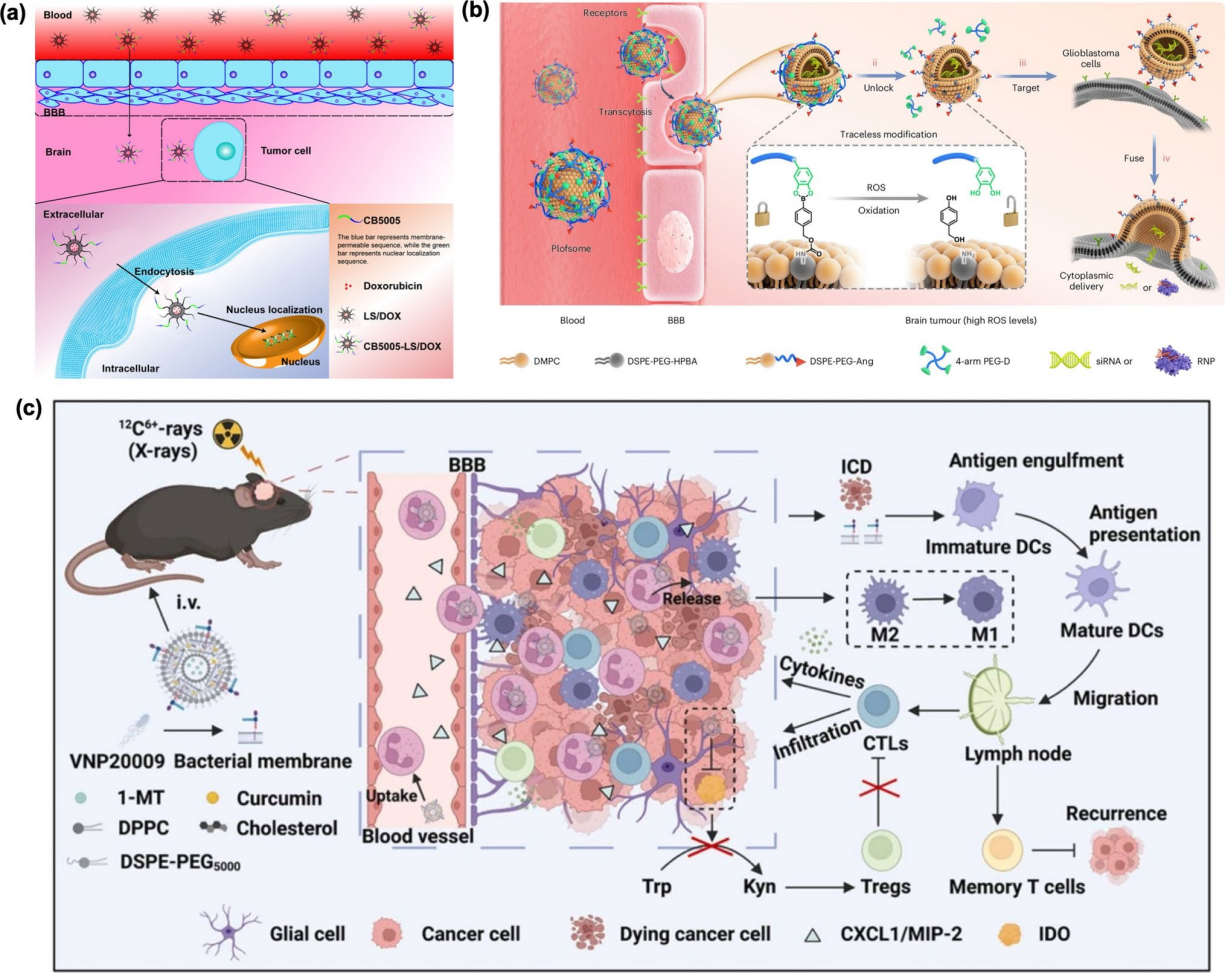
** (a)** The development of liposomes that are conjugated with cell-permeable NF-κB inhibitors provides a new strategy for treating glioma. These specialized liposomes are designed to deliver NF-κB inhibitors directly into glioma cells, enhancing their ability to cross cell membranes and effectively target the tumor [130]. **(b)** The Plofsome is created by modifying 4-arm PEG-oDPs onto the surface of Ang-modified fusogenic liposomes via a traceless ROS-cleavable linker, serving as a locking mechanism [131]. **(c)** immunoregulatory liposomes that attach to neutrophils to improve the effectiveness of carbon ion radiotherapy combined with immunotherapy for GBM treatment [132]

Zhang et al. [130] developed a novel liposome-based formulation that encapsulates DOX and is modified with the cell-penetrating peptide CB5005 (DOX@CB5005@LP). This formulation is designed for cell membrane penetration and NF-κB inhibition which targets specifically to GBM cells. In this study, treated mice with orthotopic implants of U87 GBM cells, which are widely used in GBM research (Fig. 6a).

Zhao et al. [131] introduced a novel polymer-locking fusogenic liposome (Plofsome) designed to effectively traverse the BBB and deliver short interfering RNA or CRISPR-Cas9 ribonucleoprotein complexes directly into the cytoplasm of GBM cells (Fig. 6b). The Plofsome features an integrated “lock” mechanism within the fusogenic liposome, utilizing a traceless ROS-cleavable linker that ensures fusion occurs only after the Plofsome successfully crosses the BBB and enters GBM tissue, where ROS levels are elevated. Their findings indicated that targeting MDK with Plofsomes significantly mitigated resistance to temozolomide (TMZ) and inhibited GBM growth in orthotopic brain tumor models.

Liu et al. [132] developed bacterial membrane-doped liposomes (B-Lipo) by integrating doped bacterial membranes (BM) into the phospholipid layer (Fig. 6c). These B-Lipo can be loaded with 1-methyl-D-tryptophan (1-MT) and curcumin (Cur). The B-Lipo/1-MT&Cur formulation demonstrated efficient uptake by neutrophils and the ability to cross the BBB to target brain tumors, particularly after radiation therapy (RT) when chemokines are elevated. In GBM models treated with RT, the combination of BM and the loaded drugs in B-Lipo/1-MT&Cur enhanced positive anti-tumor immune responses, such as immunogenic cell death and T cell infiltration. Additionally, they mitigated negative immune responses induced by RT, including macrophage polarization and the upregulation of indoleamine 2,3-dioxygenase (IDO), thereby fostering robust systemic innate and adaptive anti-tumor immunity. Remarkably, the synergy between carbon ion RT and B-Lipo/1-MT&Cur led to the successful eradication of most orthotopic GBM in mice while preventing recurrence, all without inducing physiological or neuropsychiatric damage. This innovative strategy showcases the potential of combining intensive RT with targeted local and systemic therapies, advancing the field of carbon ion RT and its therapeutic combinations.

The treatment with DOX@CB5005@LP showed significant enhancement in drug accumulation within tumor tissue, maximizing therapeutic efficacy. By combining the properties of liposomes and cell-penetrating peptides, this targeted approach offers a more effective strategy to address the challenges posed by GBM. Additionally, Plofsomes feature a ‘lock’ mechanism using a traceless ROS-cleavable linker, ensuring that fusion occurs exclusively after the liposomes have crossed the BBB and reached the GBM tissue. This innovative design has implications that extend beyond GBM, as the effective delivery of siRNA and CRISPR–Cas9 complexes across the BBB opens new avenues for treating other CNS disorders. Furthermore, incorporating bacterial membranes into the phospholipid layer of liposomes enhances their immunomodulatory properties. When combined with the targeted delivery of therapeutic agents, this approach represents a promising direction for future GBM therapies.

## Polymeric based formulations for brain tumor immunotherapy

Polymeric systems have played a key role in enhancing the efficacy and safety of immunotherapies. Their numerous favorable properties, including excellent biocompatibility and biodegradability, structural and compositional diversity, ease of fabrication, and high loading capacity for immune-related substances, make them particularly well-suited for brain tumor immunotherapy [133, 134]. These diverse characteristics enable polymeric systems to perform critical functions in immunotherapy, such as acting as immune stimulants, activating T cells, and serving as APCs. Immune checkpoint inhibitors (ICIs), CAR-T cells, and oncolytic viruses have demonstrated remarkable success in cancer immunotherapy, significantly improving clinical outcomes for patients. Functionalization with ligands like folate, RGD peptides, or mannose enhances the ability of polymer-based drug delivery systems to target specific receptors, increase cellular uptake, and minimize off-target effects. This modification also allows for controlled drug release and improved therapeutic efficacy. Additionally, the flexibility of polymeric systems allows for combination therapies, integrating multiple immunotherapeutic agents or delivery mechanisms, which further enhances tumor targeting and immune system activation. This versatility positions polymeric systems as a promising tool for advancing the treatment of resistant tumors [135, 136].

CTLA-4 and PD-1/PD-L1 blockade are the most widely investigated immune ICIs. The immune system relies on immune checkpoints to regulate immune responses and prevent autoimmunity, ensuring that it targets harmful pathogens or cancerous cells without attacking the body’s healthy tissues. These checkpoints act as brakes that help maintain self-tolerance. Under normal conditions, activated T cells express PD-1 as part of the immune response to recognize and eliminate abnormal or cancerous cells. However, tumor cells can evade the immune system by exploiting the PD-1/PD-L1 pathway, a natural immune regulatory mechanism [137]. Therefore, anticancer immunotherapy can be effectively achieved by using ICIs that block PD-1 or its ligand PD-L1. Another immune checkpoint CTLA-4 is another critical immune checkpoint that plays a vital role in regulating T-cell activity and maintaining self-tolerance. The anti-CTLA-4 antibodies have been developed to block the inhibitory effects of CTLA-4, leading to enhanced T cell activation and improved anti-tumor responses [138].

CTLA-4 and PD-1/PD-L1 blockade are among the most extensively studied ICIs. The immune system utilizes immune checkpoints to regulate responses and prevent autoimmunity, ensuring it targets harmful pathogens or cancerous cells while sparing healthy tissues. These checkpoints function as regulatory mechanisms that help maintain self-tolerance. Under typical conditions, activated T cells express PD-1 as part of their immune response to identify and eliminate abnormal or cancerous cells. However, tumor cells can evade immune detection by exploiting the PD-1/PD-L1 pathway, a natural immune regulatory mechanism [139]. Thus, anticancer immunotherapy can be effectively pursued by employing ICIs that block PD-1 or its ligand, PD-L1. Additionally, CTLA-4 is a crucial checkpoint that regulates T-cell activity and self-tolerance. Anti-CTLA-4 antibodies have been developed to inhibit the negative effects of CTLA-4, resulting in enhanced T-cell activation and improved anti-tumor responses [140].

**Fig. 7:**
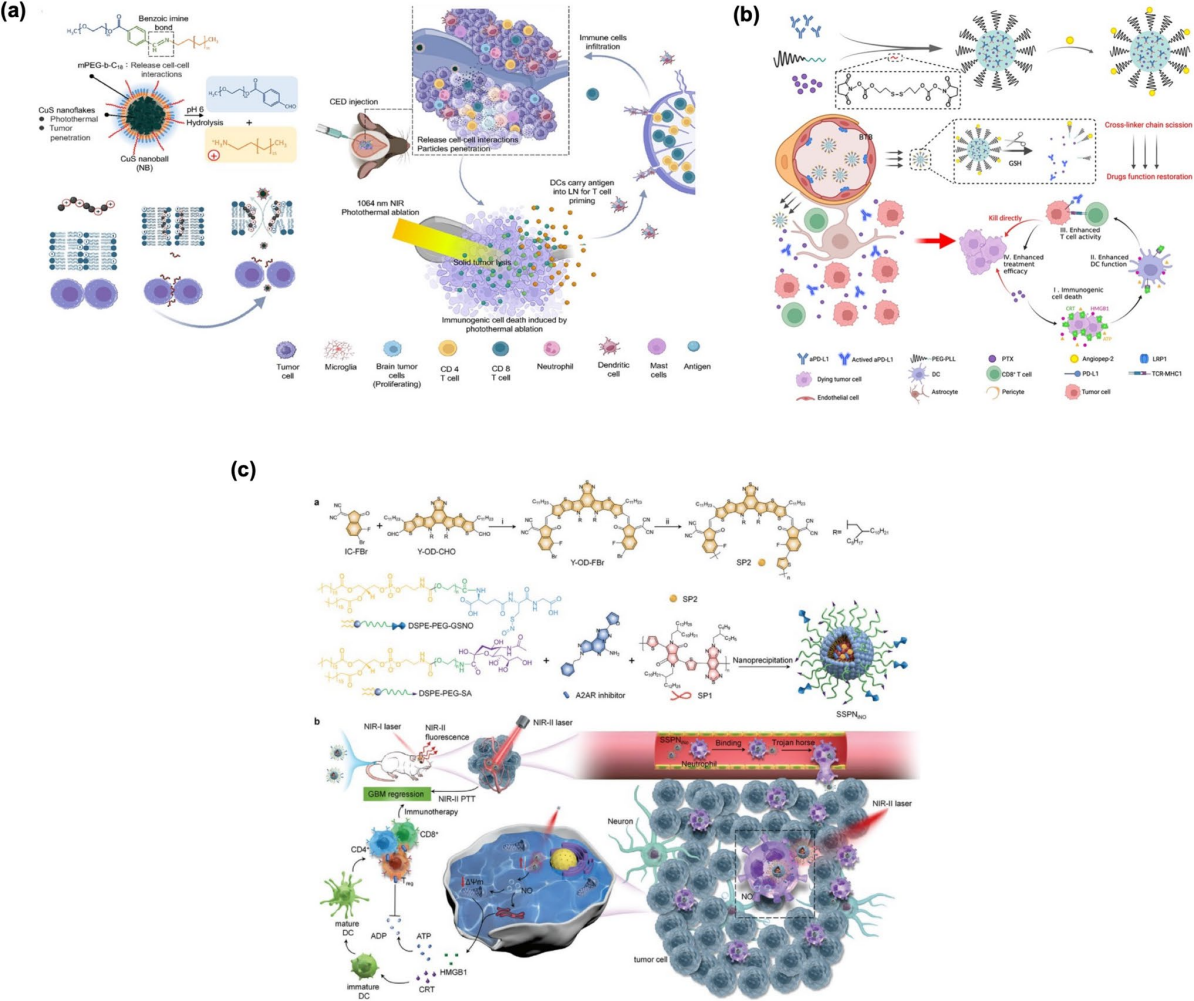
**(a)** This schematic illustrates the preparation and characteristics of the CuS nanoball (NB), consisting of membrane-disrupted polymer-wrapped CuS nanoflakes designed for brain tumor immunotherapy. The CuS NB accumulates effectively in brain tumors using a continuous positive pressure infusion technique called convection-enhanced delivery (CED). This thermal effect promotes the release of tumor-associated antigens, which are then preserved and presented to DCs, thereby enhancing the immune response mediated by CD4^+^ and CD8^+^ T cells [141]. **(b)** Redox-responsive polymer micelles are designed to co-encapsulate ICIs and chemotherapeutic agents for the treatment of GBM. The micelles respond to the redox environment within the tumor, ensuring that the drugs are released precisely when needed for optimal therapeutic action [142]. **(c)** The schematic illustrates the concept of nanotheranostics for imaging-guided PTT nitric oxide (NO) immunotherapy in orthotopic GBM. The synthesis process for SP2 and the nanotheranostics (SSPNiNO). The action for SSPNiNO-based theranostics in GBM is also depicted, highlighting how this system functions in targeting and treating the tumor effectively [143]

Nirosha et al. [141] developed a membrane-disrupted, polymer-wrapped copper sulfide (CuS) nanoflake system aimed at targeting deep brain tumors through multiple innovative strategies for effective cancer therapy (Fig. 7a). This system can penetrate deeply into brain tumors by disrupting cell-cell interactions, enabling near-infrared II (NIR-II) PTT, and capturing DCs to initiate a self-cascading immunotherapy. When subjected to low-power NIR-II irradiation, the well-distributed CuS nanoflakes generate a thermolyticSchematic illustration of features of the tight junctions in (BBB) and the concept of immune effect that facilitates tumor treatment, promoting cell apoptosis and the release of antigens.

Zhang et al. [142] developed an innovative micelle formulation, angiopep2-aPD-L1@PTX nano-micelle (A2-APM), that combines antibodies and chemotherapeutic agents for effective chemo-immunotherapy in GBM (Fig. 7b). This formulation entails crosslinking anti-PD-L1 antibodies (aPD-L1) and electrostatically binding paclitaxel (PTX) to the amino groups of polyethylene glycol-poly-L-lysine (PEG-PLL). To enhance BBB penetration, the micelles are decorated with the targeting angiopep-2 (A2) peptide. In the reductive microenvironment of GBM, the cross-linker chain breaks, triggering the release of aPD-L1 from the micelles while preserving its structure and function, which enhances the selectivity of ICB therapy for GBM. Simultaneously, the dissociation of the micelles accelerates the release of PTX in the tumor microenvironment. This rapid PTX release not only effectively inhibits GBM cell growth but also sensitizes the tumors to the PD-1/PD-L1 blockade by inducing ICD, thereby facilitating ICB therapy. This study aims to establish a bio-safe and effective chemo-immunotherapeutic strategy to address the limitations of ICB therapy in brain malignancies.

Liu et al. [143] targeted GBM using a strategy that leverages neutrophil-targeting mechanisms and advanced imaging techniques (Fig. 7c). The approach involves semiconducting polymer-based NPs that integrate second near-infrared (NIR-II) fluorescence imaging with a robust trimodal therapy strategy. This design features two semiconducting polymers: one serves as a NIR-II fluorescence probe while the other functions as a PTT conversion agent, allowing for precise imaging and targeted therapy delivery. These NPs, named SSPNiNO, are equipped with a dual therapeutic payload consisting of a heat-sensitive nitric oxide (NO) donor and an inhibitor of the adenosine 2 A receptor (A2AR), enabling a three-pronged attack on the tumor. By incorporating a neutrophil-targeting ligand on their surface, the NPs utilize a “Trojan Horse” strategy to infiltrate GBM tissues effectively. The A2AR inhibitor encapsulated within the NPs helps modulate the immunosuppressive tumor microenvironment by blocking the adenosine-A2AR pathway, which enhances the immune response against the tumor. This combined approach leads to significant tumor growth suppression in GBM models.

By fabricating these CuS nanoflakes, we have created a unique system capable of penetrating deep into brain tumors. This thermolytic efficacy not only targets tumor cells directly but also enhances the release of tumor-associated antigens, thereby fostering an environment conducive to immune activation. Further, the dual functionality, where PTX exerts direct cytotoxicity on GBM cells while simultaneously enhancing the efficacy of ICB therapy. The targeted angiopep-2 (A2) peptide on the micelle’s surface serves as a critical facilitator for BBB penetration, a major hurdle in treating brain tumors. Overall, this study represents a significant step toward addressing the limitations of ICB therapy in brain malignancies. Moreover, using neutrophils as delivery vehicles through a “Trojan Horse” mechanism highlights a novel approach to overcoming the BBB. The trimodal therapeutic action of SSPNiNO combining PTT, NO release, and immune modulation adds to the therapeutic arsenal. This combination of targeted delivery, precise imaging, and multi-faceted therapeutic action makes SSPNiNO a promising candidate for advancing GBM treatment.

## Outlook and conclusion

GBM presents one of the most formidable challenges in oncology due to its highly aggressive nature and the brain’s immunosuppressive environment. Immunotherapy, which activates the immune system to recognize and attack cancer cells, holds great promise in treating GBM. However, several obstacles hinder its effectiveness, particularly the brain’s immune privilege and the BBB. These factors prevent robust immune cell infiltration and limit the delivery of therapeutic agents, making it difficult to mount an effective immune response against the tumor. Despite the success of immunotherapies like checkpoint inhibitors in other cancers, their application in GBM has been limited due to these inherent challenges.

Recent advancements in nanotechnology offer hope in overcoming these barriers. Immune-actuated nanoparticles have shown significant potential in enhancing drug delivery by mimicking immune cell functions to penetrate the BBB. These particles can disrupt the BBB’s structure in a controlled manner, allowing therapeutic agents to reach the tumor while minimizing potential long-term damage. By integrating with various therapeutic approaches—such as chemotherapy, chemodynamic therapy, PTT, radiotherapy, and magnetotherapy—nanoparticles not only enhance the precision and efficacy of treatment but also help release tumor antigens, promoting an immune response against GBM.

Furthermore, these nanoparticle-based systems can be designed to retain autologous tumor-associated antigens and present them to dendritic cells, ensuring prolonged immune activation. This aspect is particularly important for GBM, as it can potentially stimulate an anti-tumor immune response that lasts beyond the initial treatment. By promoting antigen release and immune cell activation, nanomaterials offer a dual approach: they directly disrupt tumor growth while simultaneously enhancing the body’s natural immune defense mechanisms.

In summary, combining immunotherapy with advanced nanoparticle-based delivery systems represents a promising strategy for addressing the complexities of GBM. By overcoming the challenges posed by the brain’s immune privilege and the BBB, these innovations pave the way for more effective and targeted treatments, offering new hope for patients with this highly aggressive brain tumor.

## Discussion

Nanomaterial-based treatments for GBM have shown significant potential, with various materials offering distinct advantages. CNTs are highly effective for drug delivery and PTT therapy, as their functionalization with targeting ligands or drugs enhances their ability to cross the BBB and selectively kill GBM cells. However, issues such as toxicity, immune responses, and limited biodegradability remain challenges [58]. AuNPs, with their tunable optical and chemical properties, are used for imaging, PTT, and as radiosensitizers to enhance radiation therapy. Despite their promise, concerns regarding accumulation in off-target tissues and long-term safety must be addressed [83, 84]. Dendrimers, highly branched polymers, offer precise drug loading and functionalization, improving BBB penetration and tumor specificity. Conjugation with chemotherapeutic agents or nucleic acids further enhances their therapeutic potential, though their synthesis complexity and potential toxicity pose limitations [99, 100]. Nanogels, crosslinked hydrophilic polymer networks, provide excellent drug-loading capacity and controlled release. Functionalization with targeting ligands improves delivery specificity, though challenges in optimizing gelation properties persist [111, 113]. Liposomes, as biocompatible lipid vesicles, enable efficient drug encapsulation and delivery, with PEGylation improving their circulation time. However, their inability to release drugs in response to tumor-specific stimuli limits their efficacy [126, 127]. Polymeric nanoparticles, such as PLGA, PEG, or polycaprolactone, are widely used for drug and gene delivery due to their biocompatibility and versatility [133, 135]. These systems encapsulate chemotherapeutics or immune-modulatory agents, improving systemic circulation and therapeutic precision. However, consistent and sustained drug release without systemic toxicity remains a challenge.

Other therapeutic strategies for GBM should also be considered, including expanding beyond these established nanomaterials. Exosome-based nanocarriers are naturally occurring vesicles that can cross the BBB and deliver drugs to GBM cells with high biocompatibility. Stimuli-responsive nanocarriers release drugs responding to tumor-specific conditions, such as pH or redox levels, enhancing delivery precision [4, 5]. Hybrid nanomaterials, combining CNTs with AuNPs or dendrimers with liposomes, enable multimodal approaches, integrating imaging, chemotherapy, and PTT or PDT. Nanovaccines encapsulating tumor antigens and immune adjuvants can stimulate immune responses against GBM cells, with combinations including immune checkpoint inhibitors showing promise in preclinical models. Multifunctional nanoplatforms, integrating therapy and imaging, provide real-time treatment monitoring while delivering therapeutic agents, enhancing overall efficacy. While nanomaterials like CNTs, AuNPs, dendrimers, liposomes, nanogels, and polymers are transformative, incorporating advanced strategies such as exosome-based systems, gene therapies, and theranostic platforms could enhance GBM treatment outcomes. Addressing challenges like BBB penetration, off-target effects, and toxicity will be essential for translating these innovations into clinical practice.

Clinical trials are exploring innovative functional nanomaterials to enhance the effectiveness of brain immunotherapy (Table 3). These advanced materials include carbon nanotubes (CNTs), gold nanoparticles, dendrimers, nanogels, liposomes, and specialized polymers. Each offers unique capabilities, such as targeted delivery, controlled drug release, and improved stability in physiological conditions. By leveraging these materials, researchers aim to overcome challenges like crossing the blood-brain barrier, enhancing immune system targeting, and reducing systemic toxicity. The ultimate goal is to significantly improve therapeutic outcomes for brain-related disorders and diseases, offering a more precise and effective approach to immunotherapy.

**Table 3:** Clinical trials focused on the development of functional nanomaterials, including CNTs, gold nanoparticles, dendrimers, nanogels, liposomes, and polymers, for improving brain immunotherapy outcomes

Nanomaterial Type	Therapeutic Goal	Target Disease	Details	Stage
Au NPs	PTT + immune activation	Glioblastoma	Investigating gold nanoparticles for tumor ablation and immune system activation post-treatment.	NCT04911864 (Recruiting)
Liposomes	Enhanced drug delivery to the tumor site	Glioblastoma	Evaluating pegylated liposomal doxorubicin for better drug retention and reduced toxicity.	NCT01636267 (Completed)
Polymeric NPs	Targeted chemotherapy with albumin-bound paclitaxel	GBM	Studies the efficacy of albumin-bound nanoparticles for improved targeted chemotherapy.	NCT03020017 (Active)
PLGA NPs	Immune modulation via dendritic cell activation		PLGA nanoparticles loaded with tumor antigens to promote immune response against brain tumors	NCT02736764 (Completed)
Lipid NPs	mRNA delivery for cancer vaccine	Solid Tumors (including glioblastoma)	Testing lipid nanoparticles for delivering mRNA vaccines targeting tumor-specific antigens.	NCT04671031 (Ongoing)
Dendrimers	Targeted delivery of immunotherapeutic agents		Investigating dendrimer-based delivery systems for targeted immunotherapy in brain tumors.	NCT03354210 (Completed)
Nanogels	Immunotherapy via nanogel-based vaccine delivery		Evaluating nanogels for controlled release of immunotherapeutics in brain tumor treatment.	NCT02969944 (Completed)

## Conclusions

Recent advancements in medicine, biochemistry, protein engineering, and materials science have propelled significant progress in nanoscale targeting methods for brain tumor immunotherapeutics. However, despite these advancements, numerous challenges hinder the widespread clinical application and effective treatment of conditions like GBM. Researchers are diligently developing innovative therapeutic strategies to overcome tumor resistance and improve clinical outcomes for GBM. These strategies emphasize enhancing T cell infiltration to combat GBM effectively. Various nanomedicines are being explored to deliver synergistic combinations of medications, bolstering both innate and adaptive immune responses, thereby improving immunotherapy efficacy in targeting GBM. Although the investigation of nano-delivery systems to stimulate cellular immune responses in GBM patients is still in its early stages, it holds substantial promise for advancing immunotherapy.

## Supplementary information

Below is the link to the supplementary material.

ESM 1 (PDF 5.42 MB)
