# Supplementary material for: Advancing brain immunotherapy through functional nanomaterials
Source: Drug Deliv Transl Res. 2025 Jan 9;16(3):983–1006. doi: 10.1007/s13346-024-01778-5 (PMC12876551; doi:10.1007/s13346-024-01778-5)
Supplement: Supplementary file 1 — (PDF 5.42 MB) [file 13346_2024_1778_MOESM1_ESM.pdf]

Figure. 2 (a) mCNTs induce cell death under a rotating magnetic field. Rotating magnetic field and (mCNTs exert mechanical work and stimulation to tumor cells. [46] (b) Immunostimulatory CpG on Carbon Nanotubes Selectively Inhibits Migration of Brain Tumor Cells [49] (c) Schematic graph of CNT-based DDS in TME targeting. [50]

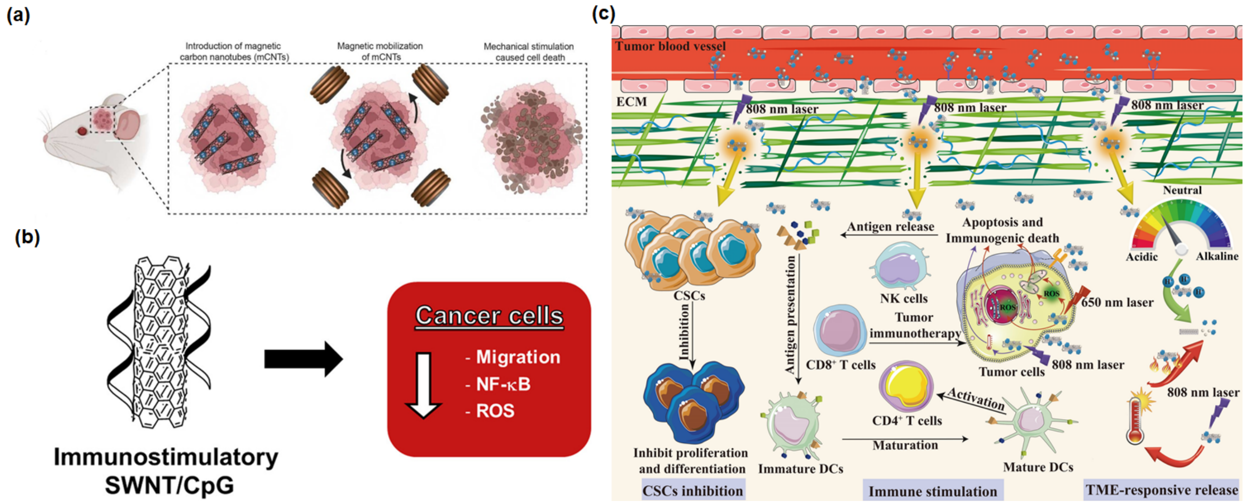

(a) Copyright Permission

Science Advances AAAS

Mechanical nanosurgery of chemoresistant glioblastoma using magnetically controlled carbon nanotubes

Author: Xian Wang, Zheyuan Gong, Tiancong Wang, Junhui Law, et al.

Publication: Science Advances

Publisher: The American Association for the Advancement of Science

Date: Mar 29, 2023

Copyright © 2023, The American Association for the Advancement of Science

Creative Commons

This is an open access article distributed under the terms of the Creative Commons CC BY license, which permits unrestricted use, distribution, and reproduction in any medium, provided the original work is properly cited.

You are not required to obtain permission to reuse this article.

(b) Copyright Permission

CCC RightsLink Sign in/Register

ACS Publications Most Trusted. Most Cited. Most Read.

Immunostimulatory CpG on Carbon Nanotubes Selectively Inhibits Migration of Brain Tumor Cells

Author: Darya Alizadeh, Ethan E. White, Teresa C. Sanchez, et al

Publication: Bioconjugate Chemistry

Publisher: American Chemical Society

Date: May 1, 2018

Copyright © 2018, American Chemical Society

PERMISSION/LICENSE IS GRANTED FOR YOUR ORDER AT NO CHARGE

This type of permission/license, instead of the standard Terms and Conditions, is sent to you because no fee is being charged for your order. Please note the following:

- Permission is granted for your request in both print and electronic formats, and translations.
- If figures and/or tables were requested, they may be adapted or used in part.
- Please print this page for your records and send a copy of it to your publisher/graduate school.
- Appropriate credit for the requested material should be given as follows: "Reprinted (adapted) with permission from {COMPLETE REFERENCE CITATION}. Copyright {YEAR} American Chemical Society." Insert appropriate information in place of the capitalized words.
- One-time permission is granted only for the use specified in your RightsLink request. No additional uses are granted (such as derivative works or other editions). For any uses, please submit a new request.

If credit is given to another source for the material you requested from RightsLink, permission must be obtained from that source.

BACK CLOSE WINDOW

(c)

CCC RightsLink

Insights on functionalized carbon nanotubes for cancer theranostics

SPRINGER NATURE

Author: Lu Tang et al
Publication: Journal of Nanobiotechnology
Publisher: Springer Nature
Date: Dec 16, 2021

Copyright © 2021, The Author(s)

Creative Commons

This is an open access article distributed under the terms of the Creative Commons CC BY license, which permits unrestricted use, distribution, and reproduction in any medium, provided the original work is properly cited.

You are not required to obtain permission to reuse this article.
CC0 applies for supplementary material related to this article and attribution is not required.

Figure. 3 (a) The schematic illustration of hierarchical targeting RVG-hybrids via an aggregation transition in the weak acidity. The magnetoelectric disassembly of RVGhybrids facilitates the penetrative delivery of dendrimers and GQDs. [65] (b) Schematic illustration of our synergistic strategy for glioblastoma treatment. [69] (c) Spiky Gold Nanoparticles, a Nanoscale Approach to Enhanced Ex Vivo T-Cell Activation. [71]

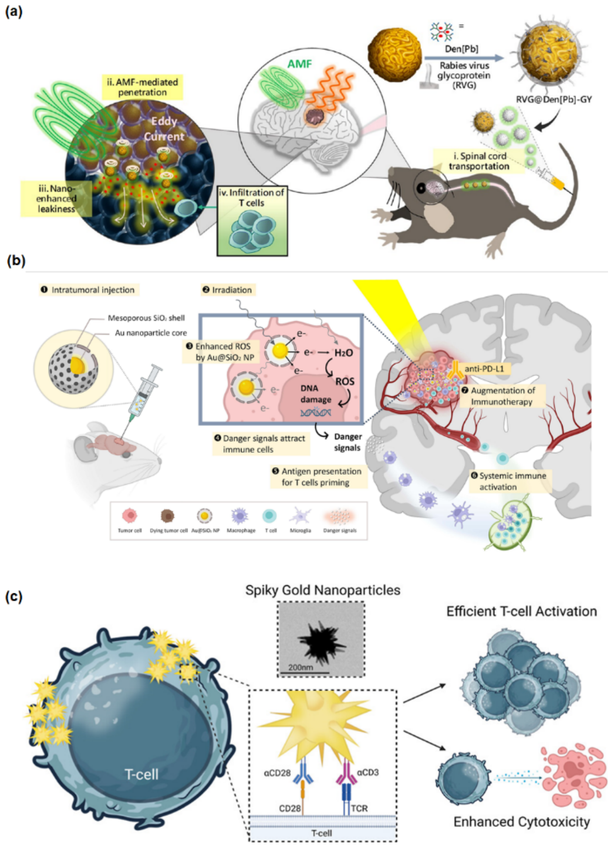

(a) Copyright Permission

(b) Copyright Permission

Removed from paper

CCC | RightsLink

BY

Different-Sized Gold Nanoparticle Activator/Antigen Increases Dendritic Cells Accumulation in Liver-Draining Lymph Nodes and CD8 T Cell Responses

ACS Publications
Most Trusted. Most Cited. Most Read.

Author: Qianqian Zhou, Yulong Zhang, Juan Du, et al
Publication: ACS Nano
Publisher: American Chemical Society
Date: Feb 1, 2016

Copyright © 2016, American Chemical Society

PERMISSION/LICENSE IS GRANTED FOR YOUR ORDER AT NO CHARGE

This type of permission/license, instead of the standard Terms and Conditions, is sent to you because no fee is being charged for your order. Please note the following:

- Permission is granted for your request in both print and electronic formats, and translations.
- If figures and/or tables were requested, they may be adapted or used in part.
- Please print this page for your records and send a copy of it to your publisher/graduate school.
- Appropriate credit for the requested material should be given as follows: "Reprinted (adapted) with permission from {COMPLETE REFERENCE CITATION}. Copyright {YEAR} American Chemical Society." Insert appropriate information in place of the capitalized words.
- One-time permission is granted only for the use specified in your RightsLink request. No additional uses are granted (such as derivative works or other editions). For any uses, please submit a new request.

If credit is given to another source for the material you requested from RightsLink, permission must be obtained from that source.

BACK CLOSE WINDOW

(c) Copyright Permission

CCC | RightsLink

BY

Spiky Gold Nanoparticles, a Nanoscale Approach to Enhanced Ex Vivo T-Cell Activation

ACS Publications
Most Trusted. Most Cited. Most Read.

Author: Fatemeh Esmaeili, Yuhao Leo Wu, Zongjie Wang, et al
Publication: ACS Nano
Publisher: American Chemical Society
Date: Aug 1, 2024

Copyright © 2024, American Chemical Society

PERMISSION/LICENSE IS GRANTED FOR YOUR ORDER AT NO CHARGE

This type of permission/license, instead of the standard Terms and Conditions, is sent to you because no fee is being charged for your order. Please note the following:

- Permission is granted for your request in both print and electronic formats, and translations.
- If figures and/or tables were requested, they may be adapted or used in part.
- Please print this page for your records and send a copy of it to your publisher/graduate school.
- Appropriate credit for the requested material should be given as follows: "Reprinted (adapted) with permission from {COMPLETE REFERENCE CITATION}. Copyright {YEAR} American Chemical Society." Insert appropriate information in place of the capitalized words.
- One-time permission is granted only for the use specified in your RightsLink request. No additional uses are granted (such as derivative works or other editions). For any uses, please submit a new request.

If credit is given to another source for the material you requested from RightsLink, permission must be obtained from that source.

BACK CLOSE WINDOW

Figure.4 (a) Systemic dendrimer delivery of triptolide to tumor-associated macrophages improves anti-tumor efficacy and reduces systemic toxicity in glioblastoma. [78] **(b)** Glycosylation of PAMAM dendrimers significantly improves tumor macrophage targeting and specificity in glioblastoma. [79] (c) Dendrimer-Entrapped Gold Nanoparticles Boosts Efficient Tumor Immunotherapy. [80]

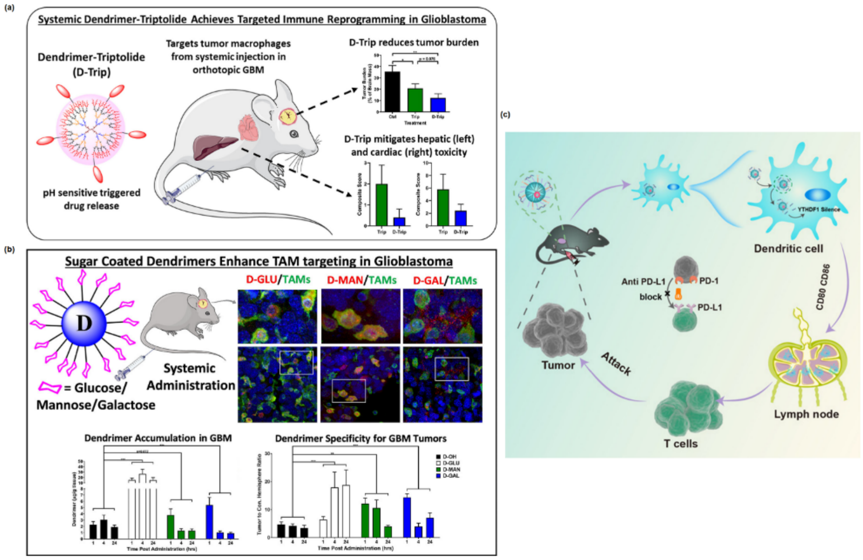

(a) Copyright Permission

(b) Copyright Permission

(c) Copyright Permission

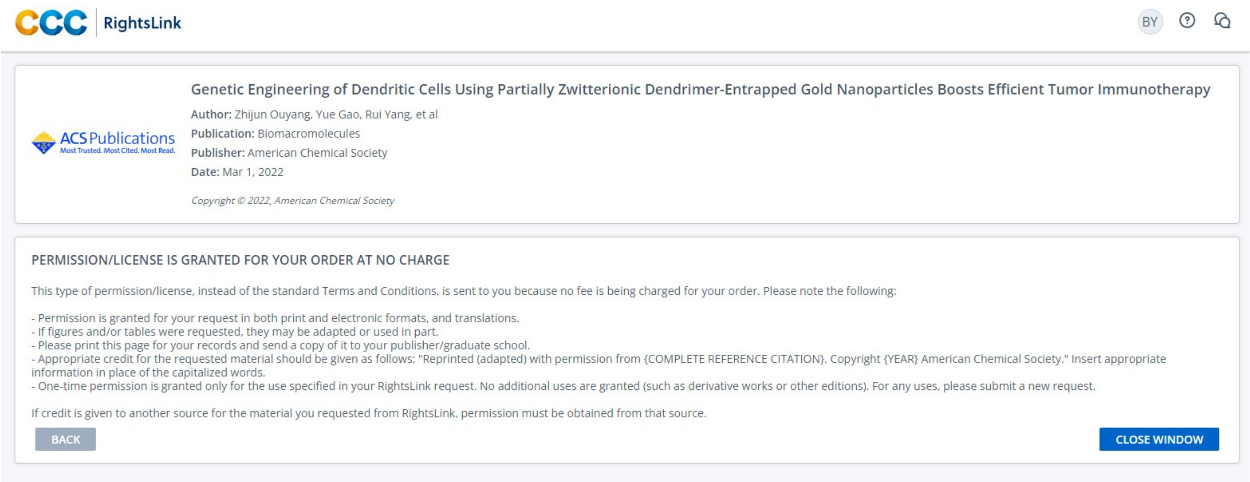
RightsLink

Genetic Engineering of Dendritic Cells Using Partially Zwitterionic Dendrimer-Entrapped Gold Nanoparticles Boosts Efficient Tumor Immunotherapy

Author: Zhijun Ouyang, Yue Gao, Rui Yang, et al
Publication: Biomacromolecules
Publisher: American Chemical Society
Date: Mar 1, 2022

Copyright © 2022, American Chemical Society

PERMISSION/LICENSE IS GRANTED FOR YOUR ORDER AT NO CHARGE

This type of permission/license, instead of the standard Terms and Conditions, is sent to you because no fee is being charged for your order. Please note the following:

- Permission is granted for your request in both print and electronic formats, and translations.
- If figures and/or tables were requested, they may be adapted or used in part.
- Please print this page for your records and send a copy of it to your publisher/graduate school.
- Appropriate credit for the requested material should be given as follows: "Reprinted (adapted) with permission from {COMPLETE REFERENCE CITATION}. Copyright {YEAR} American Chemical Society." Insert appropriate information in place of the capitalized words.
- One-time permission is granted only for the use specified in your RightsLink request. No additional uses are granted (such as derivative works or other editions). For any uses, please submit a new request.

If credit is given to another source for the material you requested from RightsLink, permission must be obtained from that source.

BACK CLOSE WINDOW

Figure. 5 (a) In situ vaccination with single-dose NvIH reduced TME immunosuppression, enhanced TME antitumor immune milieu, and elicited systemic antitumor immunity, resulting in robust immunotherapy of large poorly immunogenic tumors with abscopal effect. [90] (b) Schematic illustration of bioresponsive doxorubicin-based mannose nanobackpack for cancer immunotherapy by enhancing ICD induction. (A) Drugs that we tested for making DOX-based nanogels with high drug loading including docetaxel (DOC), paclitaxel (PTX), irinotecan (CPT-11), gemcitabine (GEM), oxaliplatin (OXA), chlorin e6 (Ce6), purpurin

18 (P18), indocyanine Green (ICG), 10-hydroxy camptothecin (HCPT), 5-fluorouracil (5-FU), 5-aminolevulinic acid (5-ALA) and mannose. (B) The synthesis process of DOX-based nanogel and simplified mechanism mediated chemo-immunotherapy to inhibit tumor growth by up-regulation immunogenic cell death. [91] (c) Schematic illustration of injectable adhesive catalytic nanoreservoir (CN) as an antigen reservoir for the enhancement of immunotherapy.a) CN containing manganese dioxide (MnO 2) and catechol-functionalized magnetic metal organic framework (cMOF) as agents to activate release andcapture tumor-associated antigens (TAAs). b) The hyperthermia and chemodynamic therapy (CDT) via Mn $^{2+}$ for redox reactions promoted cancer cell apoptosis and release TAAs. In situ-gel systems promote the retention of antigen release to reach a continuous immune stimulation and suppressing the tumor metastasis at tumor site. [92]

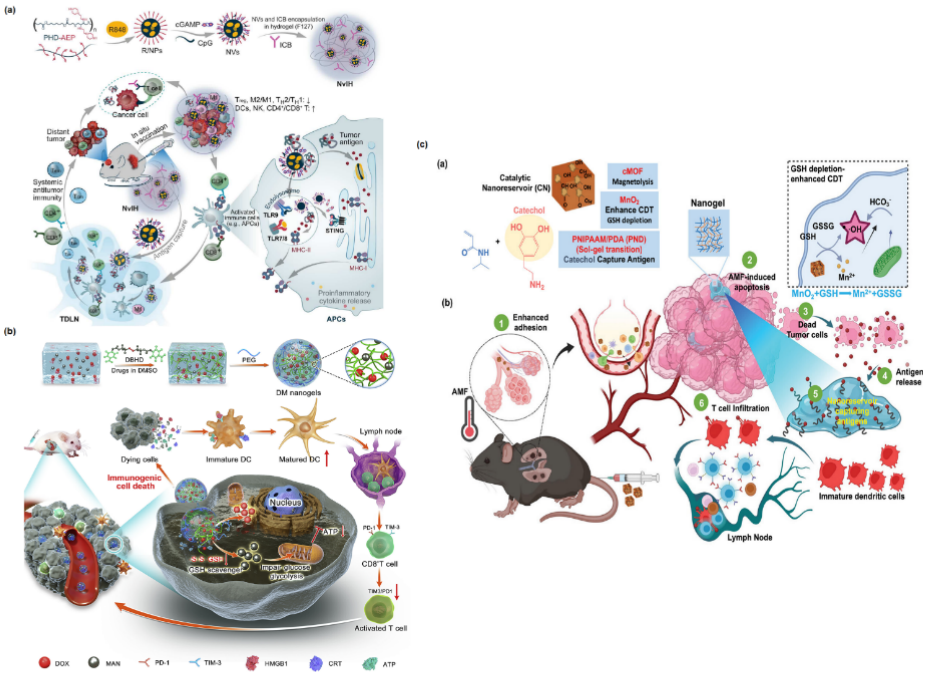

(a) Copyright Permission

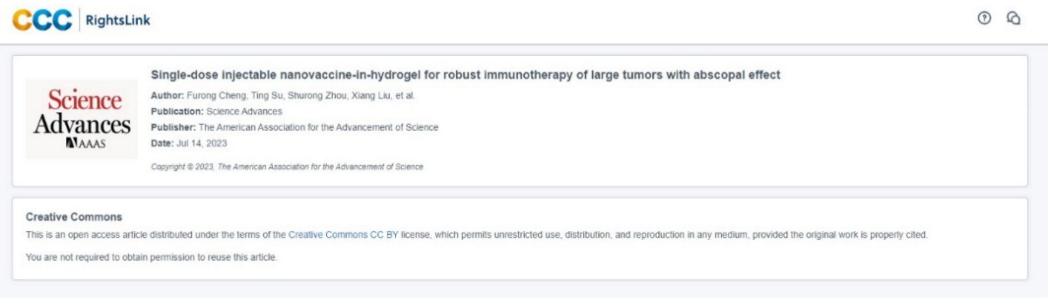
RightsLink

Single-dose injectable nanovaccine-in-hydrogel for robust immunotherapy of large tumors with abscopal effect

Author: Furong Cheng, Ting Su, Shurong Zhou, Xiang Liu, et al

Publication: Science Advances

Publisher: The American Association for the Advancement of Science

Date: Jul 14, 2023

Copyright © 2023, The American Association for the Advancement of Science

Creative Commons

This is an open access article distributed under the terms of the Creative Commons CC BY license, which permits unrestricted use, distribution, and reproduction in any medium, provided the original work is properly cited.

You are not required to obtain permission to reuse this article.

(b) Copyright Permission

(c) Copyright Permission

Figure. 6 (a) Cell-permeable NF-κB inhibitor-conjugated liposomes for treatment of glioma. [97] (b) 4-arm PEG-oDPs act as the lock and are modified onto the surface of the Ang-modified fusogenic liposome using a traceless ROS-cleavable linker to obtain the Plofsome. (i) Plofsomes are stabilized by 4-arm PEG-oDPs to form and maintain a non-fusogenic state. (ii) After entering the brain, Plofsomes are stimulated by overexpressed ROS in tumour tissues, resulting in detachment of 4-arm PEG-oDPs and becoming fusogenic. (iii) and (iv) Plofsomes target and fuse with the GBM cells by Ang-LRP-1 receptor recognition, releasing the cargoes into the GBM cell cytoplasm. [98] (c) Immunoregulatory liposomes hitchhiking on neutrophils for enhanced carbon ion radiotherapy-assisted immunotherapy of glioblastoma. [99]

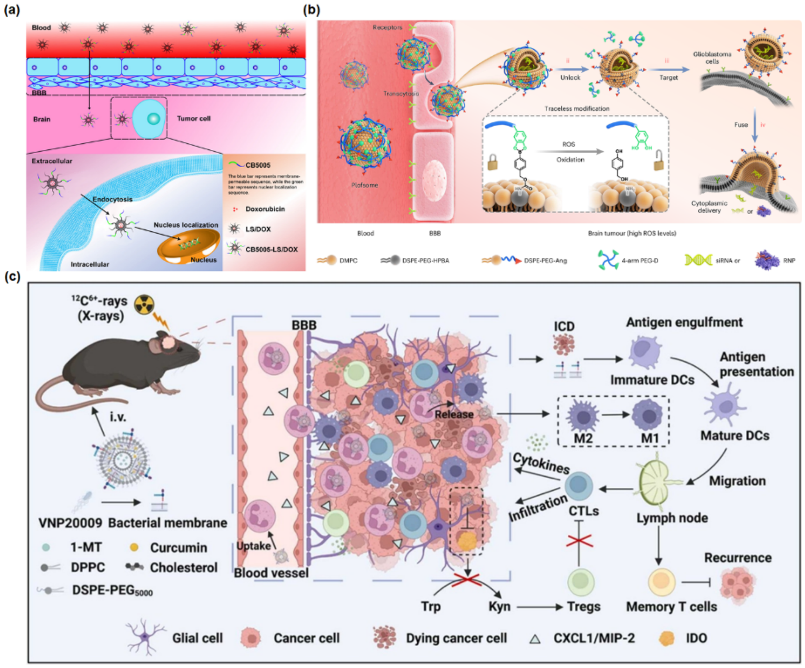

(a) Copyright permission
(b) Copyright permission
(c) Copyright permission

Figure. 7 (a) Schematic illustration of preparation and features of the CuS nanoball (NB) composed of membrane-disrupted polymer-wrapped CuS nanoflakes for brain immunotherapy. (a) The disruption of the benzoic-imine bond in mPEG-b-C18 on CuS NB enhances tumor permeability, reaching deep brain tumors by releasing cell–cell interactions under weak acidic conditions. (b) CuS NB is efficiently accumulated in

brain tumors through continuous positive pressure infusion of CED. Membrane disruption-mediated tumor penetration and low-power NIR II irradiation (0.8 W/cm2) resulted in CuS nanoflakes generating intense NIR II-generated heat deep within the tumor, promoting antigen release. This process preserves autologous tumor-associated antigens and presents them to dendritic cells, amplifying CD4+ and CD8+ T cell-mediated immune responses. [106] (b) Redox-responsive polymer micelles co-encapsulating immune checkpoint inhibitors and chemotherapeutic agents for glioblastoma therapy. [107] (c) Scheme of nanotheranostics for imaging-guided photothermal-NO-immunotherapy of orthotopic GBM. a) Synthesis of SP2 and nanotheranostics (SSPNiNO). i) Pyridine, CHCl3, 65 °C, 30 min; ii) thiophene-tin, Pd2(dba)3, P(o-tol)3, toluene, 110 °C, 72 h. b) Mechanism scheme of SSPNiNO-based theranostics of GBM. [108]

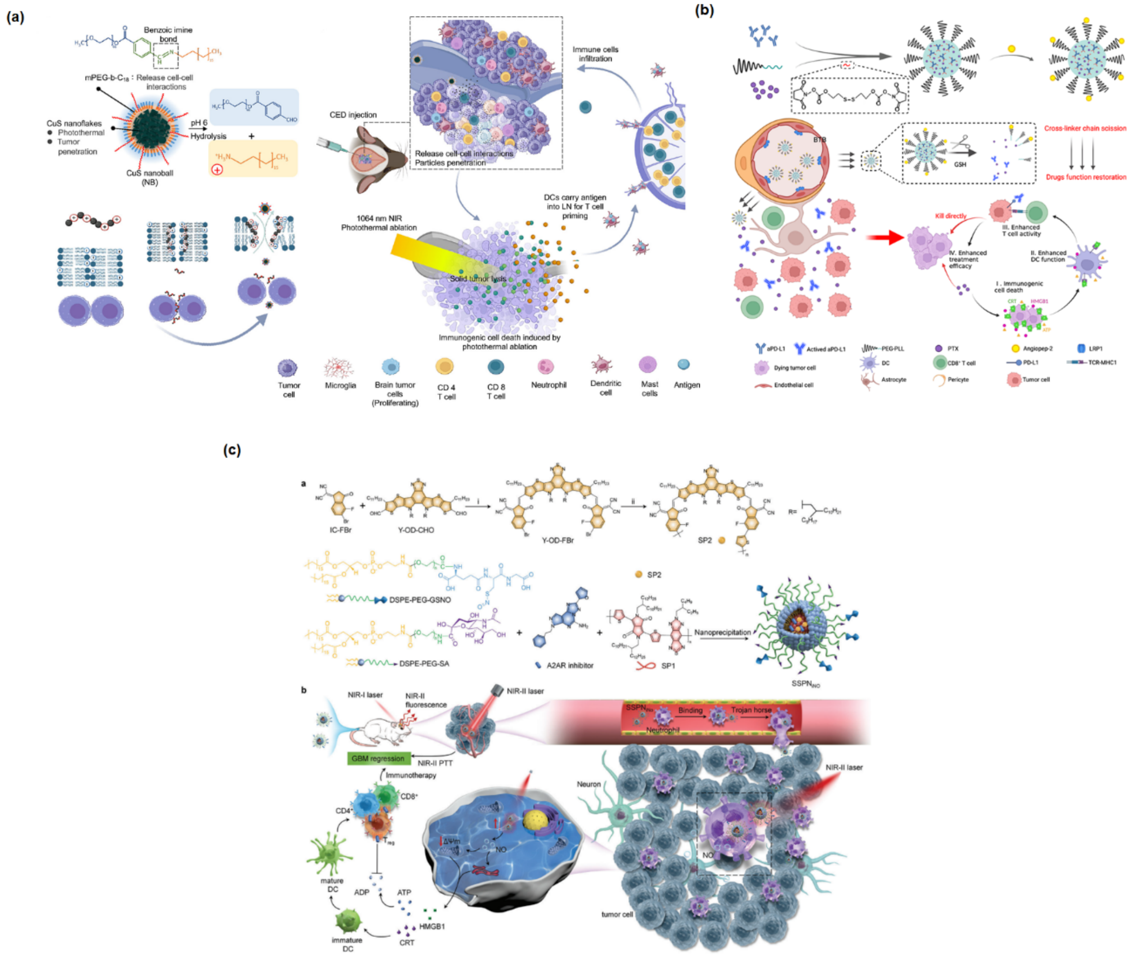

(a) Copyright permission

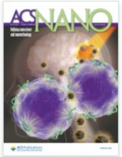

ACS Nano
Cite this: *ACS Nano* 2024, 18, 28, 18712−18728

https://doi.org/10.1021/acsnano.4c06183
Published July 2, 2024

Copyright © 2024 The Authors. Published by American Chemical Society. This publication is licensed under CC-BY 4.0 .

Get e-Alerts

License Summary*

You are free to **share** (copy and redistribute) this article in any medium or format and to **adapt** (remix, transform, and build upon) the material for any purpose, even commercially within the parameters below:

Creative Commons (CC): This is a Creative Commons license.

Attribution (BY): Credit must be given to the creator.

Article Views
1890

Altmetric
12

(b) Copyright permission

RightsLink

SPRINGER NATURE

Redox-responsive polymer micelles co-encapsulating immune checkpoint inhibitors and chemotherapeutic agents for glioblastoma therapy

Author: Zhiqi Zhang et al
Publication: Nature Communications
Publisher: Springer Nature
Date: Feb 6, 2024

Copyright © 2024, The Author(s)

Creative Commons

This is an open access article distributed under the terms of the Creative Commons CC BY license, which permits unrestricted use, distribution, and reproduction in any medium, provided the original work is properly cited.

You are not required to obtain permission to reuse this article.
To request permission for a type of use not listed, please contact Springer Nature

(c) Copyright permission

RightsLink

WILEY

Neutrophil-Targeting Semiconducting Polymer Nanotheranostics for NIR-II Fluorescence Imaging-Guided Photothermal-NO-Immunotherapy of Orthotopic Glioblastoma

Author: Jiansheng Liu, Danling Cheng, Anni Zhu, et al
Publication: Advanced Science
Publisher: John Wiley and Sons
Date: Aug 19, 2024

© 2024 The Author(s). Advanced Science published by Wiley-VCH GmbH

Open Access Article

This is an open access article distributed under the terms of the Creative Commons CC BY license, which permits unrestricted use, distribution, and reproduction in any medium, provided the original work is properly cited.

You are not required to obtain permission to reuse this article.

For an understanding of what is meant by the terms of the Creative Commons License, please refer to Wiley's Open Access Terms and Conditions.

Permission is not required for this type of reuse.

Wiley offers a professional reprint service for high quality reproduction of articles from over 1400 scientific and medical journals. Wiley's reprint service offers:

- Peer reviewed research or reviews
- Tailored collections of articles
- A professional high quality finish
- Glossy journal style color covers
- Company or brand customisation
- Language translations
- Prompt turnaround times and delivery directly to your office, warehouse or congress.

Please contact our Reprints department for a quotation. Email corporatesaleseurope@wiley.com or corporatesalesusa@wiley.com or corporatesalesDE@wiley.com.
